# Tinnitus Suppression by Intracochlear Electrical Stimulation in Single Sided Deafness – A Prospective Clinical Trial: Follow-Up

**DOI:** 10.1371/journal.pone.0153131

**Published:** 2016-04-25

**Authors:** Remo A. G. J. Arts, Erwin L. J. George, Miranda Janssen, Andreas Griessner, Clemens Zierhofer, Robert J. Stokroos

**Affiliations:** 1 Department of ENT/Head and Neck Surgery, Maastricht University Medical Center, MHeNS School for Mental Health and Neuroscience, Maastricht, the Netherlands; 2 Department of Methodology and Statistics, Maastricht University, CAPHRI School for Public Health and Primary Care, Maastricht, the Netherlands; 3 Institute of Mechatronics, University of Innsbruck, Innsbruck, Austria; University of Regensburg, GERMANY

## Abstract

**Introduction:**

Earlier studies show that a Cochlear Implant (CI), capable of providing intracochlear electrical stimulation independent of environmental sounds, appears to suppress tinnitus at least for minutes. The current main objective is to compare the long-term suppressive effects of looped (i.e. repeated) electrical stimulation (without environmental sound perception) with the standard stimulation pattern of a CI (with environmental sound perception). This could open new possibilities for the development of a “Tinnitus Implant” (TI), an intracochlear pulse generator for the suppression of tinnitus.

**Materials and Methods:**

Ten patients with single sided deafness suffering from unilateral tinnitus in the deaf ear are fitted with a CI (MED-EL Corporation, Innsbruck, Austria). Stimulation patterns are optimized for each individual patient, after which they are compared using a randomized crossover design, with a follow-up of six months, followed by a 3 month period using the modality of patient’s choice.

**Results:**

Results show that tinnitus can be suppressed with intracochlear electrical stimulation independent of environmental sounds, even long term. No significant difference in tinnitus suppression was found between the standard clinical CI and the TI.

**Conclusion:**

It can be concluded that coding of environmental sounds is no requirement for tinnitus suppression with intracochlear electrical stimulation. It is therefore plausible that tinnitus suppression by CI is not solely caused by an attention shift from the tinnitus to environmental sounds. Both the standard clinical CI and the experimental TI are potential treatment options for tinnitus. These findings offer perspectives for a successful clinical application of the TI, possibly even in patients with significant residual hearing.

**Trial Registration:**

TrialRegister.nl NTR3374

## Introduction

Tinnitus aurium, meaning ringing of the ears is the phantom sensation of sound. It is a frequent symptom of hearing dysfunction, affecting about 50 million people in the United States and an estimated 70 million in the European Union [1]. For some of them it can be extremely burdensome and affect daily life. Furthermore, the economic burden of tinnitus to society is substantial with an annual tinnitus related health care cost per patient of €1,544 on average in the Netherlands [2]. The exact underlying mechanism is not completely known, but it is plausible that tinnitus has a central origin that is triggered by auditory deprivation as a maladaptive homeostatic compensation mechanism [3]. It has long been known that auditory deprivation can induce phantom sounds when subjects spend time in complete silence in a sound-proof booth [4]. More recently, it has been reported that continuous use of an earplug can also lead to the perception of tinnitus [5].

Due to plasticity, reversing auditory deprivation by electrical stimulation should suppress the tinnitus theoretically. Although intracochlear electrical stimulation seems to be a potential treatment option for tinnitus there is no cure available yet. Research in the past decade show tinnitus suppression in bilateral and unilateral deafness using Cochlear Implants (CIs) [6,7]. It is not yet clear if the processing of speech, that is, the perception of environmental sounds is a requirement of these observed effects. The question arises if it is possible that similar effects on tinnitus, or even optimization of these effects may be achieved by meaningless, but highly controlled, intracochlear electrical stimulation. Previous studies, including preliminary results of this study, show short-term (i.e. for minutes) tinnitus reduction using electrical stimulation that does not encode environmental sounds [8–10]. This preliminary work [10] was performed in order to find the optimal stimulation characteristics for tinnitus suppression using electrical stimulation independent of environmental sounds. These preliminary results showed a tinnitus reduction during standard clinical CI rehabilitation while the CI surgery itself had no positive or negative effect on tinnitus. Furthermore, the original tinnitus loudness restored after one week of CI deactivation. Optimal stimulation using meaningless electrical stimulation for short-term tinnitus suppression was observed to be subject-specific. In order to determine whether meaningless chronic intracochlear electrical stimulation is a viable treatment option for people with extremely burdensome tinnitus, long-term effects need to be investigated. The primary goal of this study is to investigate the long term (i.e. up to three months) effects of intracochlear electrical stimulation, that does not encode environmental sounds, on tinnitus and compare these effects with the effects obtained using standard clinical CI.

## Materials and Methods

This study is a continuation of the previously published preliminary work. This study reports findings on the same participants used for the preliminary work [10], but evaluates long-term (i.e. up to three months) effects of meaningless intracochlear electrical stimulation instead of the short term (i.e. for minutes) effects reported previously and also controls for possible placebo effects. For a detailed description of the materials and methods used, see this previously published preliminary work [10]. A concise but adequate description follows below.

### Subjects

Ten adults with Single Sided Deafness (SSD) were included with an audiometric hearing threshold of at least 70 dB HL Pure Tone Average (PTA); averaged across 0.5, 1 and 2 kHz in one ear. Their contralateral ear had a moderate to normal audiometric hearing threshold (PTA better than 50 dB HL). In the preliminary work of this study they received a cochlear implant (CI) in the deaf ear in order to suppress their unilateral tinnitus, localized in the deaf ear. Inclusion criteria for implantation were: chronic, continuous and moderate-to-severe tinnitus that was stable for at least one year. Moderate-to-severe tinnitus was diagnosed as a tinnitus loudness of at least 7.0 on a Visual Analogue Scale (VAS) with a range from 0 to 10, a Tinnitus Handicap Inventory (THI) [11] score of at least 38 and/or a Tinnitus Questionnaire (TQ) [12] score of at least 42. Exclusion criteria were medical contraindications for cochlear implantation, diagnosed objective tinnitus, psychiatric disorders, depression and use of antidepressant medication.

### Design

**Fig 1:**
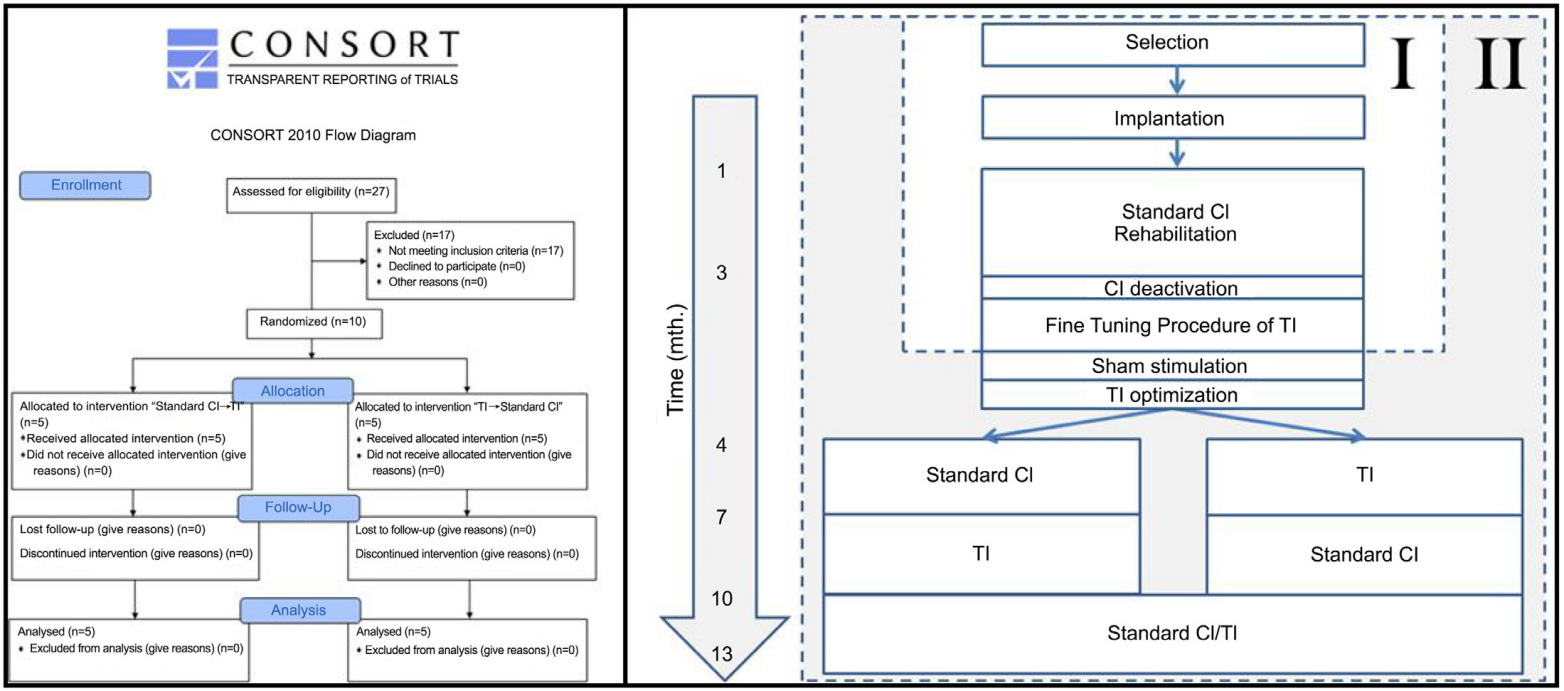
Flowcharts. Left: CONSORT 2010 Flow Diagram, right: schematic overview of the used study design indicating the preliminary work (I) which is published elsewhere [10] and the current follow-up study (II). CI: cochlear implant; TI: tinnitus implant.

Fig 1 shows a schematic presentation of the study design. Here we report on the results of the follow-up of this study, see Arts et al. for a detailed description of the preliminary work [10].

This report focused on the long-term effects of intracochlear electrical stimulation on tinnitus. In the preliminary work, the short-term effects of looped (i.e. repeated) intracochlear electrical stimulation on tinnitus were investigated after a period of standard CI rehabilitation. The standard CI rehabilitation was performed prior to the fine tuning procedure of the Tinnitus Implant (TI) in order to optimize and stabilize the dynamic range for electric hearing.

After the fine tuning procedure of the TI a sham stimulation was performed for one week to control for possible placebo effects. Here, a sub-threshold stimulation level of less than 0.5 nC per electrode was used to avoid warning signals of the processor and remote control (single-blinded procedure). Subjects were informed that the stimulation applied was sub-threshold and therefore inaudible, but that the current level was at a level at which significant tinnitus suppression was plausible. Subsequently, for each subject the four most efficient stimulation patterns during the fine tuning procedure were used on a daily basis (for details, see Arts et al. [10]). Subjects were asked to rank their tinnitus loudness and stimulus comfort at the end of each day. Each of the four stimulation patterns were tested for two full days to choose the single most convenient pattern, which was consequently utilized during the randomized crossover design. Here, either the processor was first programmed for three months as a standard clinical CI in order to perceive environmental sounds after which the processor was switched for three months to function as a looped pattern generator (TI) or vice versa. Finally, subjects were allowed to choose, based on their experiences, to use their processor either as a speech processor or as a pattern generator for another three-month follow-up period. At the end of the trial, it was allowed to fit the processor with both modalities, each programmed in a different program bank.

### Device and software

In the preliminary work of this study subjects were implanted with a MED-EL cochlear implant system (MED-EL Corporation, Innsbruck, Austria), consisting of a CONCERTO implant and the OPUS2 processor. The OPUS2 was fitted as a speech processor (CI) with standard clinical software, Maestro version 4.1.2 and 6.0 using MED-EL’s Diagnostic Interface Box II or MAX programming interface while the OPUS2 was programmed as a pattern generator (TI) using customized software (Inst. of Mechatronics, Innsbruck, Austria) in Matlab version 7.11.0 (R2010b) (The Mathworks, Natick, MA, USA) using MED-EL’s Diagnostic Interface Box II.

### Outcome measurements

#### Tinnitus

A VAS method, psychoacoustic measurements (pitch and loudness matching), duration of Residual Inhibition (RI) and questionnaires (THI and TQ) were used as tinnitus specific outcome measures.

For the VAS method, the subjects marked how they perceived tinnitus loudness, amount of discomfort, effect on life and extent of problems due to the tinnitus, each on a 10 cm bar [13]. Values were accurate to one decimal place.

Tinnitus pitch and loudness were acoustically measured, using headphones (Telephonics, TDH-39P), via the contralateral ear. Subjects were asked to concentrate on the predominant pitch of their tinnitus. Tinnitus pitch matching (PM) was performed, after one week of sham stimulation, prior to the crossover design and both after one and three months of standard CI and TI. Furthermore, tinnitus PM was performed after one and three months during the implant use of choice following the crossover design. Pure tones, 1/3 octave narrow band noises and warble tones within the 250–8000 Hz range (for the center frequency in case of narrow band noises) and white noise were presented using a three-Alternative Forced Choice method. Separate “runs” were applied for either octave and interoctave frequencies from low-to-high frequency and from high-to-low frequency respectively [9].

Tinnitus loudness was acoustically measured in dB Sensation Level (dB SL). Subjects were asked to indicate whether their tinnitus was louder or softer than the tinnitus pitch matched stimulus. A two-down, one-up adaptive staircase rule [14] with step sizes of 5 dB for rough determination and 1 dB for precise determination were used. Starting point was 15 dB SL. Tinnitus Loudness Matching (TLM) was performed, after one week of sham stimulation, prior to the crossover design and both after one and three months of standard CI and TI. Finally, TLM was performed after one and three months during implant use of choice following the crossover design.

In case of subjective tinnitus suppression by intracochlear electrical stimulation the RI was measured. RI is the length of persistence in the reduction of tinnitus after the electrical stimulation was stopped [15].

The degree of handicap due to the perceived tinnitus was evaluated with the internationally validated THI after one week of sham stimulation, prior to the crossover design and both after one and three months of standard CI and TI. Furthermore, the THI was filled out after one and three months during use of choice following the crossover design. The THI quantifies the effect of tinnitus on the patient’s emotions and daily activities using 25 items, each answered with No (0 points), Sometimes (2 points) or Yes (4 points). A higher score indicates a more severe handicap [11,16].

Tinnitus distress was evaluated using the Dutch validated TQ [12,17] after one week of sham stimulation, prior to the crossover design and both after one and three months of standard CI and TI. The TQ was also filled out after one and three months during implant use of choice following the crossover design. The questionnaire consists of 52 questions, with a maximum score of 84. Higher scores indicate more severe levels of distress.

#### Quality of Life

The Health Utilities Index Mark III (HUI mark III) was used to estimate health-related quality of life [18,19]. The HUI mark III consists of 17 questions of which 12 questions are used to compute eight dimensions: vision, hearing, speech, emotion, pain/complaints, ambulation, dexterity and cognition. Possible overall utility scores range from -0.36 (the all-worst health state) to 0.00 (dead) to 1.00 (perfect health). The single-attribute utility score for the hearing dimension was obtained from question 3 and 4 and varies between 0.00 and 1.00. The HUI mark III was filled out prior to the CI-surgery, after three months of standard CI and TI and after three months during implant use of choice following the crossover design.

#### Depression

To measure the behavioral manifestation of depression the Beck Depression Inventory (BDI) was used [20,21]. The BDI is a 21-item questionnaire that explicitly stresses the aspects of depression. Each item can be scored from 0 to 3, with a total score of 0–13 for minimal depression, 14–19 for light depression, 20–28 corresponds to moderately serious depression, and finally, a score of 29 or more corresponds to serious depression. The BDI was filled out prior to the CI-surgery, after three months of standard CI and TI and after three months during the implant use of choice following the crossover design.

#### Speech perception

Speech perception in silence, speech perception in noise and results from the Speech, Spatial and Quality of hearing questionnaires will be reported elsewhere.

### Statistics

Statistics were performed with IBM SPSS Statistics, version 20, and *p* values smaller than 0.05 were, unless otherwise reported, considered statistically significant.

#### Crossover design

In order to assess the effectiveness of the TI on tinnitus, the outcomes obtained during TI were compared to the outcomes obtained during standard CI. Two-sided Mann-Whitney U tests (Exact) were performed to determine possible carryover effects, period effects and treatment effects. A carryover effect in a crossover design occurs when the effects of one or both interventions during the first active period have a residual biological effect during the second active period. Testing for possible carryover effects is important because no washout period was used in the current methodological design. The power of the test to detect carryover effects is limited. Therefore, *p* values smaller than 0.10 were considered statistically significant [22]. Period effects show a clear preference for the former or latter period and could bias treatment effects. Treatment effects were studied to determine the effectiveness of the TI in relation the CI.

#### Effectiveness of intracochlear electrical stimulation on tinnitus suppression

Using this methodological design, the current study was able to assess both a possible placebo effect as well as the effect of intracochlear electrical stimulation on tinnitus. Non-parametric Friedman tests for multiple comparisons were performed prior to post hoc testing with two-sided Wilcoxon signed-rank tests. A statistically significant Friedman test allows us to perform post hoc testing. In order to assess the placebo effect, baseline outcomes (before implantation) were compared to the outcomes after one week of sham stimulation. The effect of intracochlear electrical stimulation on tinnitus was assessed by comparing baseline outcomes to the outcomes obtained at the end of the follow-up.

### Ethics

This study was designed and conducted in accordance with the declaration of Helsinki. Ethics approval was obtained from the Ethics Committee of Maastricht University/academic hospital Maastricht (approval No. NL38789.068.11). An independent Data Safety Monitoring Board was used to oversee the safety of the included subjects. All subjects gave written informed consent before participation.

## Results

During this clinical trial, subject 2 struggled as she adjusted to the new sound provided by the implant. This was partially due to her noisy and stressful daily environment which included taking care of her two young children and her mother who passed away during the trial. Subject 8 had a stressful period with her partner and child which made wearing her hearing prosthesis complicated. Nevertheless, none of the included subjects dropped out prematurely and no serious adverse events that can be related to the treatment were observed.

### Stimulation patterns

Following TI optimization of the marked potential stimulation patterns in the preliminary work[10] on a daily basis, Table 1 shows the stimulation patterns, one for each subject, which were subjectively scored as the most convenient stimuli. This pattern was used during TI-stimulation in the current longitudinal study. In eight out of the ten included subjects the electrical stimulation was presented on the tinnitus pitch matched electrode. In five of them the stimulation was presented at this single electrode, in one subject the stimulation was presented on the tinnitus pitch matched electrode together with the two adjacent electrodes and in the other two subjects the stimulation was presented on all the available electrodes. Furthermore, eight out of ten subjects preferred a cathodic first charge-balanced biphasic stimulation. During the crossover design it was allowed to reduce the applied current level of the looped stimulation patterns by consultation of our tertiary otologic practice in case of for example tinnitus reduction or in order to improve stimulus comfort.

**Table 1:** Subject-specific stimulation during TI-use. Charge-balanced biphasic stimulation in monopolar mode.

Subject	Pattern	Electrode(s)	Amplitude Modulation	Polarity (first A/C)	Stimulation rate (pps/ channel)	Mean pulse width/ channel (μs)	Final mean maximum charge value/ channel (nC)
1	5	10 (PM elec.)	Fixed	C	200	69	12.1
1*	41	10 (PM elec.)	Random	C	5000	85	9.1
2	App. 2	1 & 2	Fixed	C	200	60	3.2
3	28	All	Random	C	750	79	5.5
4	48	9 (PM elec.)	Sine wave	C	PM	74	3.3
5^‡^	40	8 & 9 (two adjacent elec. of PM elec.)	Random	C	4918^□^	80	6.1
6	47	11 (PM elec.)	Sine wave	C	5000	62	6.6
7	13	6 (PM elec.)	Fixed	C	200	69	10.4
8	27	7 (PM elec.)	Random	C	750	65	8.0
9	App. 2	7–9 (PM elec. + two adjacent elec.)	Random	A	PM	84	7.1
10	App. 2	All	Fixed	A	750	88	2.5

A: anodic, App.: appendix; C: cathodic

*: change of stimulation pattern after 1 month due to suboptimal effect on tinnitus

^‡^: partial insertion with two extracochlear electrodes

^□^: limited due to the overall stimulation rate

The most convenient stimulation patterns were obtained from preliminary results [10]. Furthermore, results obtained from subject 4 were additionally presented as a case report [23].

### Crossover design

The included subjects were equally distributed in a randomized order between the two treatment arms, consisting of standard CI followed by TI or vice versa. Table 2 shows the results obtained during the crossover design. Here, descriptive statistics include mean score, standard deviation (SD), median and interquartile range (IQR) of both TI and CI. Results were obtained after one month and three months of treatment. No carryover effects were obtained. A statistically significant period effect was found only for the BDI-score after three months of treatment in which the depression-score in period 1 was higher compared to period 2, regardless of the treatment arm. No significant treatment effects were observed for the tested variables after both one month and three months of treatment.

**Table 2:** Differences between TI and CI, investigated in a crossover design.

		Implant function		Carryover effect (Exact)	Period effect (Exact)	Treatment effect (Exact)
		TI N = 10	CI N = 10			
1 month treatment						
Averaged VAS-score	Mean	4.20	3.69	*p* = 0.548	*p* = 0.167	*p* = 0.246
	SD	2.41	2.25			
	Median (IQR)	3.35 (2.68–6.95)	3.15 (2.00–5.80)			
TLM	Mean	13.30	12.90	*p* = 0.730	*p* = 0.397	*p* = 0.881
	SD	13.70	9.53			
	Median (IQR)	7.50 (4.75–20.00)	12.00 (5.75–18.00)			
TQ	Mean	29.60	29.70	*p* = 0.841	*p* = 0.175	*p* = 0.770
	SD	13.06	11.84			
	Median (IQR)	30.00 (19.25–38.25)	27.00 (23.50–38.50)			
THI	Mean	38.40	34.80	*p*>0.999	*p* = 0.802	*p* = 0.056
	SD	15.77	14.88			
	Median (IQR)	40.00 (25.00–44.50)	38.00 (21.50–44.50)			
3 months treatment						
Averaged VAS-score	Mean	4.53	3.79	*p* = 0.968	*p* = 0.500	*p* = 0.389
	SD	2.80	2.58			
	Median (IQR)	3.40 (2.40–7.63)	3.50 (1.55–6.63)			
TLM	Mean	12.50	13.00	p>0.999	*p* = 0.683	*p* = 0.857
	SD	10.34	8.84			
	Median (IQR)	9.50 (3.75–20.25)	11.00 (7.00–17.75)			
TQ	Mean	32.10	28.30	*p* = 0.595	*p* = 0.389	*p* = 0.183
	SD	12.81	16.63			
	Median (IQR)	30.00 (22.50–34.75)	23.50 (13.75–43.25)			
THI	Mean	40.40	35.00	*p* = 0.524	*p* = 0.730	*p* = 0.151
	SD	16.49	14.52			
	Median (IQR)	40.00 (25.00–52.00)	31.00 (22.00–46.50)			
BDI	Mean	6.70	6.10	*p* = 0.889	*p* = 0.024 (period 1 > period 2)	*p* = 0.333
	SD	4.08	2.92			
	Median (IQR)	7.50 (3.75–10.00)	6.00 (4.75–7.50)			
HUI Mark III overall	Mean	0.715	0.698	*p* = 0.738	*p* = 0.206	*p* = 0.738
	SD	0.210	0.203			
	Median (IQR)	0.745 (0.625–0.865)	0.720 (0.535–0.865)			
HUI Mark III hearing	Mean	0.801	0.773	*p* = 0.738	*p* = 0.397	*p*>0.999
	SD	0.240	0.221			
	Median (IQR)	0.930 (0.480–1.000)	0.860 (0.480–1.000)			

VAS: Visual Analogue Scale, TLM: Tinnitus Loudness Match, TQ: Tinnitus Questionnaire, THI: Tinnitus Handicap Inventory, BDI: Beck Depression Inventory, HUI: Health Utilities Index, SD: standard deviation, IQR: interquartile range, TI: tinnitus implant, CI: cochlear implant.

#### Treatment effect of intracochlear electrical stimulation

Although no statistically significant differences on tinnitus related outcomes, depression and quality-of-life between TI and CI could be found (Table 2), subject 9 was the only one who chose for TI following the crossover design. All other subjects preferred CI, probably because of the audiological advantages compared to TI which will be reported elsewhere. For consistency, the current report presents the same tinnitus related outcome measures as in the preliminary results of this study [10].

**Fig 2:**
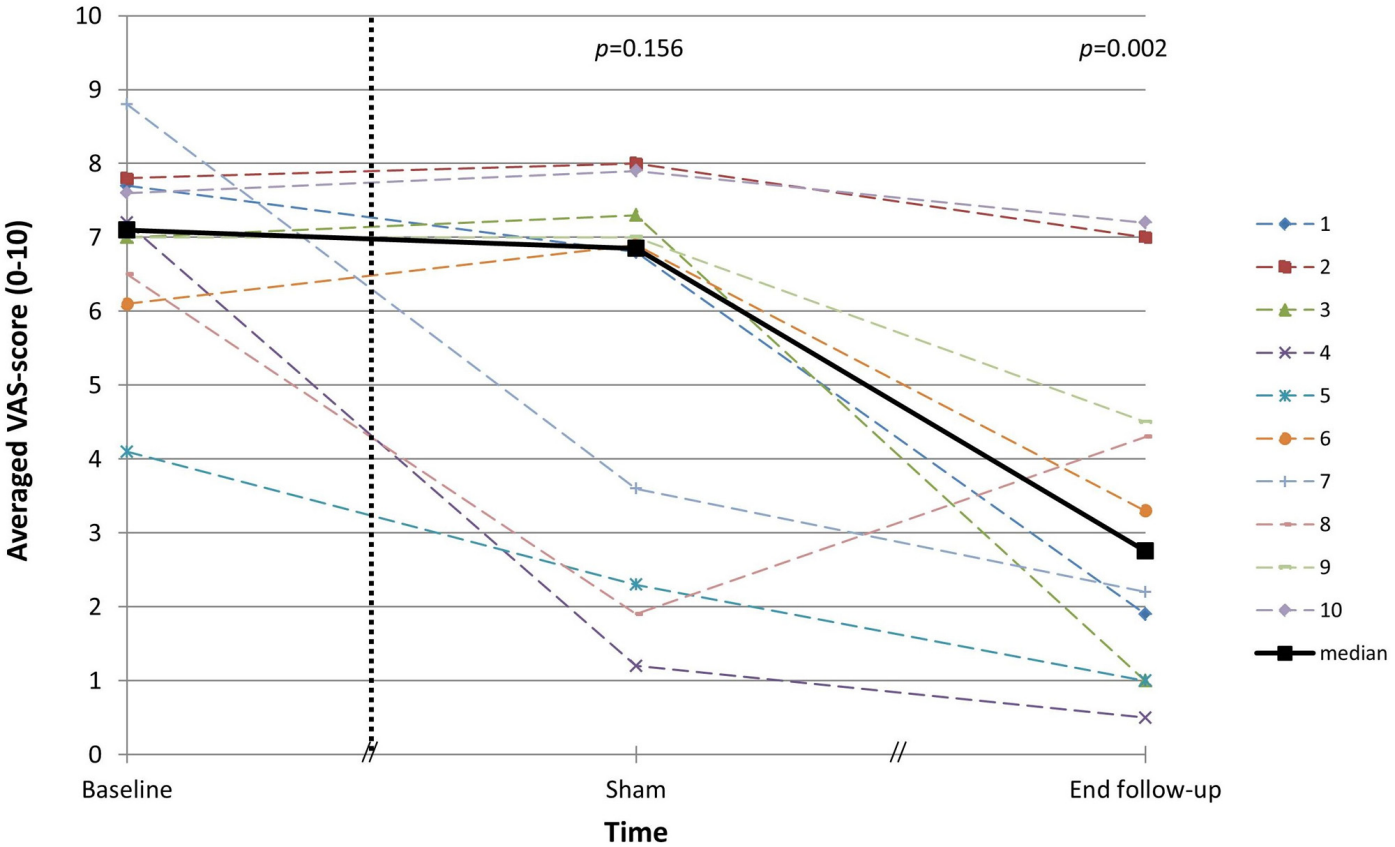
The averaged score on a Visual Analogue Scale (0–10) on perceived tinnitus loudness, amount of discomfort, effect on life and extent of problems due to the tinnitus. Individual results and median are shown at baseline, after one week of sham stimulation and at the end of the follow-up.

Fig 2 shows the averaged VAS-scores of perceived tinnitus loudness, amount of discomfort, effect on life and extent of problems due to the tinnitus prior to surgery, after one week of sham stimulation and at the end of the follow-up, i.e., after three months of implant use of choice. The Friedman test was statistically significant (*p* = 0.001). Post-hoc tests show no significant difference in averaged VAS-score between baseline and sham stimulation (*p* = 0.156). A statistically significant reduction of the averaged VAS-score was observed at the end of the follow-up compared to baseline (*p* = 0.002).

**Fig 3:**
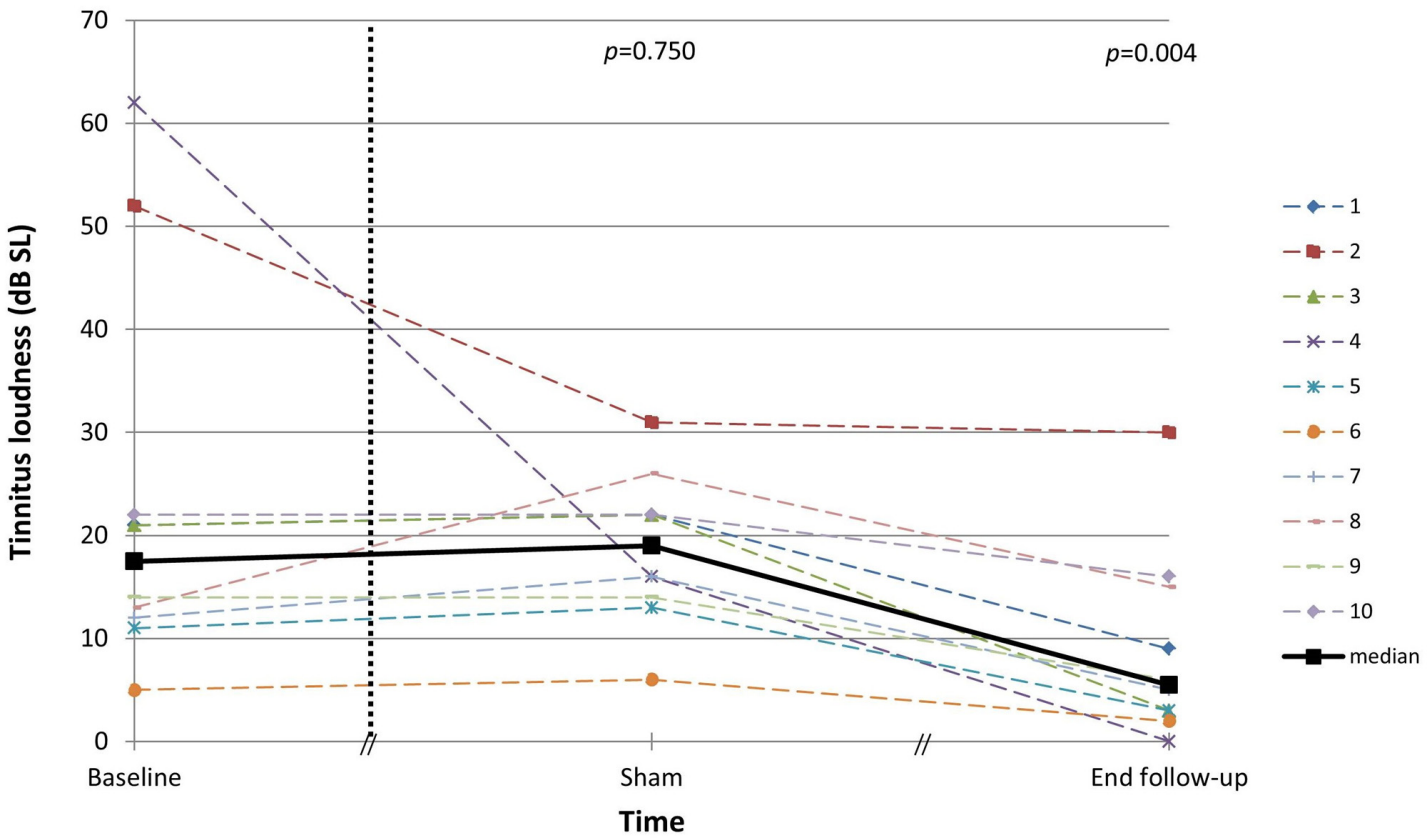
Tinnitus loudness matched via the contralateral normal hearing ear at baseline, after one week of sham stimulation and at the end of the follow-up. Individual results and median are shown.

The tinnitus loudness, matched via acoustic stimulation to the contralateral normal hearing ear, was expressed in dB SL prior to surgery, after one week of sham stimulation and at the end of the follow-up (Fig 3). The Friedman test was statistically significant (*p*<0.001). Again, no significant difference in tinnitus loudness was obtained between baseline and sham stimulation (*p* = 0.750) while the difference between baseline and the end of the follow-up was statistically significant (*p* = 0.004).

**Fig 4:**
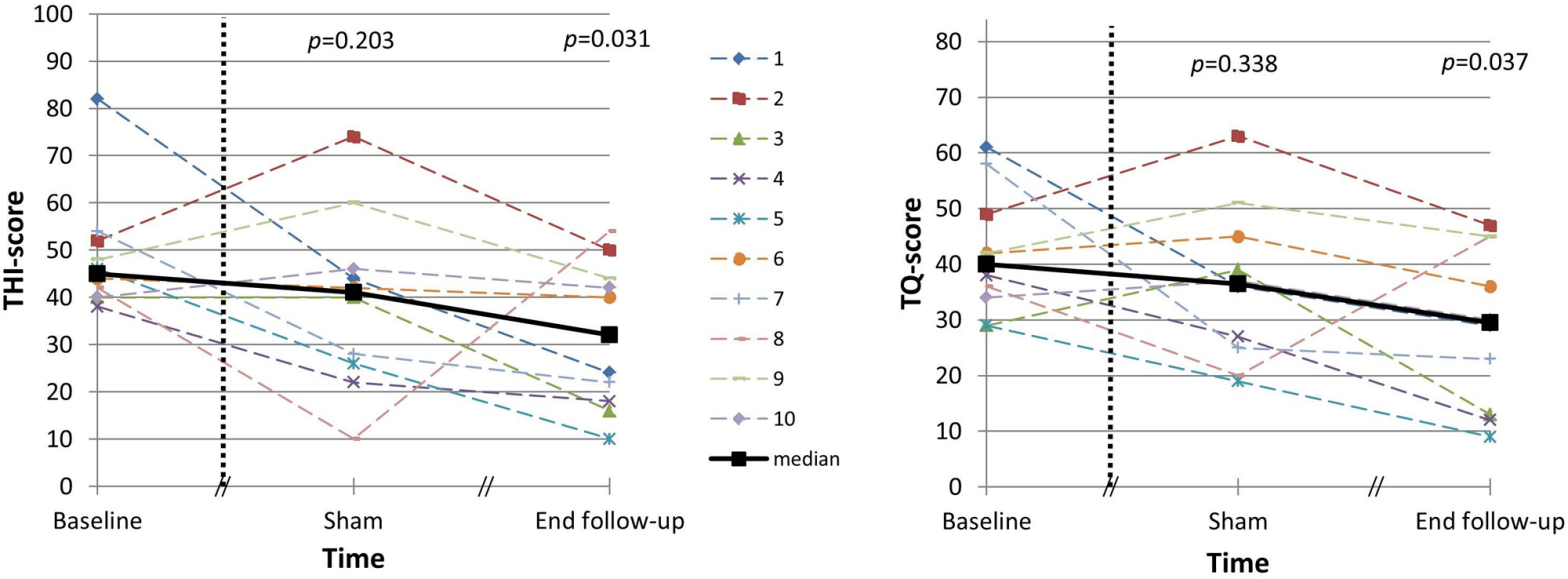
Subject-specific results of the Tinnitus Handicap Inventory (left graph) and Tinnitus Questionnaire (right graph) at baseline, after one week of sham stimulation and at the end of the follow-up.

The total THI and TQ scores prior to surgery, after one week of sham stimulation and at the end of the follow-up are shown in Fig 4. The Friedman tests were statistically significant (*p* = 0.018 and *p* = 0.026 respectively). No significant differences in tinnitus handicap (THI) and tinnitus distress (TQ) were obtained between baseline and sham stimulation (*p* = 0.203 and *p* = 0.338 respectively). Significant reductions were obtained between baseline and after completing this clinical trial (*p* = 0.031 and *p* = 0.037 respectively).

**Fig 5:**
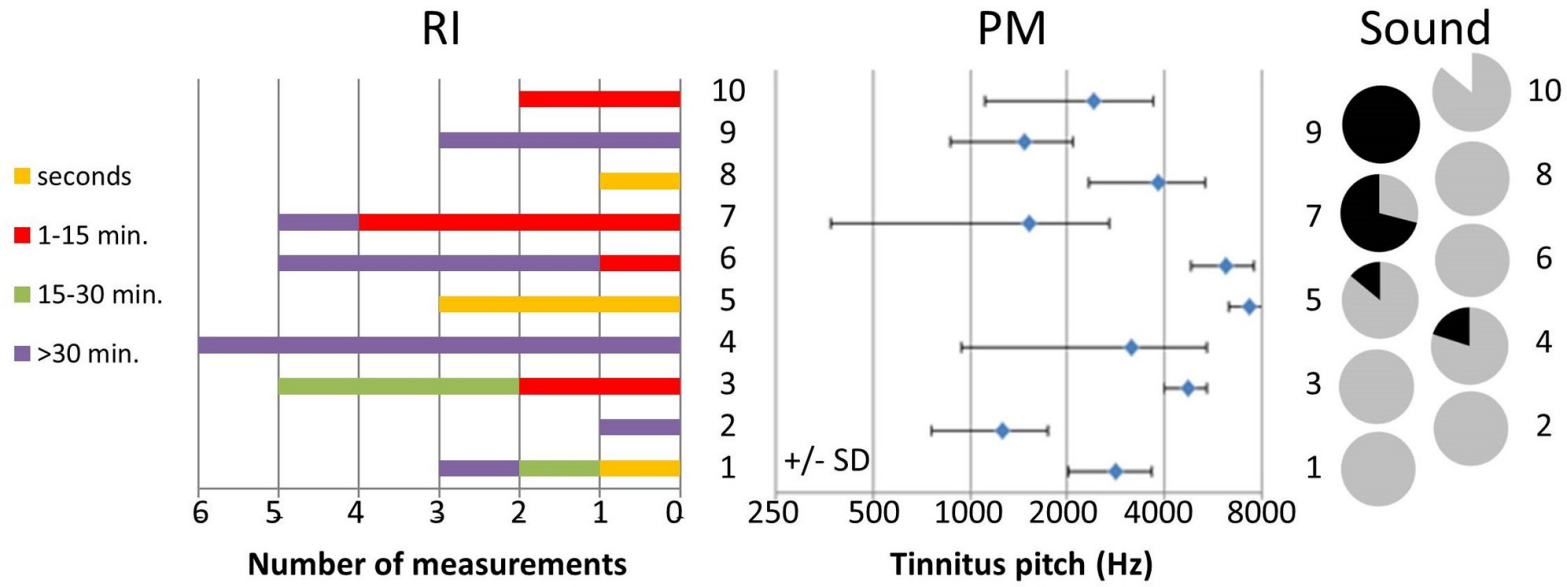
Subject-specific Residual Inhibition (left graph), tinnitus pitch (middle graph) and type of tinnitus perception (right graph) during the crossover design and the three-month follow-up period during modality-use of patient’s choice. The Y-axis represents the individual subjects. The percentages of the types of tinnitus perceptions were color-coded in the individual pie charts; black for pure tones, grey of narrow band noises and white for white noise.

In each subject the RI was measured at least once. Of the 34 times the RI was measured during this trial, in 15% of the cases the reduction of tinnitus persisted for seconds. For 26% of the cases the reduction persisted between one and 15 minutes and in another 12% of the cases the reduction continued between 15 and 30 minutes. The majority of the measured residual inhibitions (47%) persisted for more than 30 minutes. All six of the residual inhibitions measured in subject 4 persisted for more than 30 minutes while all three of the residual inhibitions measured in subject 5 continued for seconds (Fig 5).

Furthermore, Fig 5 shows the results obtained from tinnitus pitch matching during the crossover design and the three-month follow-up period during modality-use of patient’s choice (middle and right graph). For five subjects the tinnitus was perceived in all the measurements as a narrow band noise while the tinnitus perceptions in the other subjects fluctuated over time between pure tones, narrow band noises and white noise. No warble tones were perceived.

Table 3 shows the results obtained from the BDI, the HUI Mark III overall utility score and the HUI Mark III score for the hearing dimension prior to the surgery and at the end of the follow-up. Descriptive statistics include mean score, SD, median and IQR. No statistically significant difference in depression and quality of life, for both the overall utility score as well as the single-attribute utility score for the hearing dimension, could be found between baseline and the end of the follow-up (*p* = 0.563, *p* = 0.148 and *p*>0.999 respectively).

**Table 3:** Depression and utility scores.

		Baseline	End follow-up	Wilcoxon signed-rank test (Exact)
BDI	Mean	6.90	7.20	*p* = 0.563
	SD	1.85	6.32	
	Median (IQR)	7.00 (5.00–8.25)	6.00 (3.25–10.75)	
HUI Mark III overall	Mean	0.809	0.727	*p* = 0.148
	SD	0.173	0.300	
	Median (IQR)	0.845 (0.738–0.955)	0.725 (0.695–0.925)	
HUI Mark III hearing	Mean	0.868	0.906	*p*>0.999
	SD	0.212	0.164	
	Median (IQR)	1.000 (0.765–1.000)	1.000 (0.860–1.000)	

BDI: Beck Depression Inventory, HUI: Health Utilities Index, SD: standard deviation, IQR: interquartile range.

At the end of this clinical trial it was allowed to fit the processor with both standard clinical CI and TI, with each modality programmed in a different program bank. Using this combination of implant functions subjects were able to select the preferred modality depending on the daily situation by using their remote control. Six out of the ten included subjects (60%) chose for a combination of speech processing (CI) and looped stimulation (TI) while four subjects (40%) chose only for speech processing.

## Discussion

### Observations

Our study shows no statistically significant difference between looped intracochlear electrical stimulation (TI) and standard clinical CI on tinnitus outcome measures during the crossover design. There was also no significant difference between treatments found on the BDI and HUI Mark III. However, a period effect was observed for the BDI-scores that could probably bias the analysis of the treatment effect during this crossover design.

All used tinnitus outcome measures showed a statistically significant reduction at the end of the follow-up compared to baseline while no significant difference was found between baseline and after one week of sham stimulation. This treatment did not result in complete tinnitus suppression (except for 1 subject), but the reduction in tinnitus was significant. The BDI-scores and HUI Mark III utility scores at the end of the follow-up were not statistically different from baseline. Furthermore, RI could often be measured during the trial and ranged from a few seconds to more than 30 minutes. The tinnitus was most often perceived as an 1/3 octave narrow band noise. Finally, no serious adverse events that can be related to neither the standard clinical CI nor the TI were observed.

### Comparison of CI and TI

There is no literature available (with the exception of two case reports, of which one anecdotally [23,24]) on the long-term effectiveness of intracochlear electrical stimulation independent of environmental sounds on tinnitus. The current study shows no statistically significant difference between looped intracochlear electrical stimulation and standard clinical CI in any of the analyzed outcome measures after both one month and three months of treatment in the used crossover design. Therefore, speech perception appears to be no requirement for tinnitus suppression using intracochlear electrical stimulation. However, the results on the depression scale need to be interpreted with caution since a period effect was detected that could influence a possible treatment effect. The preference in the majority of subjects of using the CI in relation to the TI during the trial (only 1 subject preferred using the TI following the crossover design) is plausibly declared by the audiological advantages using the CI compared to the TI.

No statistically significant differences in the health-related quality of life were found both during the crossover design as well as in the comparison between baseline and at the end of the follow-up. Therefore it is assumed that no response shift biased the analyses of the tinnitus outcome measures [25]. Response shift is the phenomenon that subjects change their internal standards when they experience changes in health.

### Effectiveness of intracochlear electrical stimulation on tinnitus suppression

It is generally known that intracochlear electrical stimulation by using standard clinical CI suppresses tinnitus in both the bilateral severe to profound deaf population as well as the SSD population [26–32]. Our results are consistent with these findings. It is worth mentioning that the significant reduction in the tinnitus loudness matched via the contralateral ear is a subtle addition to the existing literature. Nevertheless, the current study shows no significant improvement of the health-related quality of life after cochlear implantation which is consistent with a previous study using the HUI Mark III after 6 months of CI use in eleven adult subjects with unilateral deafness [33]. However, these results are not in agreement with a previous German study using the disease-specific Nijmegen Cochlear Implant Questionnaire in the bilateral deaf population [34]. This inconsistency could probably be declared by the difference in questionnaire used or the difference in the studied population. The HUI Mark III was initially chosen since Maes and colleagues found that this utility measure was preferred in a tinnitus population, compared to the EuroQol-5D [19]. A hearing handicap and chronic tinnitus may be associated with emotions such as helplessness and depressive symptoms. Nevertheless, the statistically significant reduction of tinnitus after cochlear implantation did not result in a significant reduction of the depression score. This is consistent with a previous study [34] and was initially expected because the absence of depression complaints was one of the criteria for subject inclusion [10]. Moreover, as far as the authors known this is the first study that included a sham stimulation in an attempt to control, although using a single-blinded design, for possible placebo effects.

### Variation between subjects

The heterogeneity of auditory deprivation could declare the considerable variation in effectiveness between subjects. The current consensus is that tinnitus is the result of maladaptive plasticity in the central auditory pathway as a result of auditory deprivation [10]. Therefore, it is plausible that tinnitus could be reversible by restoring auditory stimulation. To be effective it is assumed to be necessary to bypass the cause of auditory deprivation which can occur at various positions along the auditory pathway. As intracochlear electrical stimulation directly stimulates the cochlear nerve and bypasses the transduction at the level of the hair cells, a more central origin of auditory deprivation is presumably outside the range of this treatment option. The suboptimal results obtained in for example subject 2 could therefore possibly be explained by a more central pathology due to the HELLP-syndrome, although no evidence was found for this argument [10]. This assumption is in accordance with the high effectiveness of electrical stimulation on tinnitus found in the subjects with a history of Morbus Menière (subject 3 and 7). Furthermore, both subject 2 and subject 8 were in a stressful period during the trial which could possibly have hampered the effectiveness on tinnitus suppression. This emphasizes the importance of an appropriate selection procedure.

### Limitations

The study reported was a pilot study, and outcomes could have been victim to potential methodological limits, starting with the small sample size which is mainly the result of the significant costs related to cochlear implantation. It is therefore desirable that alternatives of the relatively expensive standard clinical CI will become under investigation. The current study could possibly contribute to investigate these alternatives.

It is also important to note in the interpretation of the results that the test for period effect in the crossover design described above has low sensitivity. Especially for small crossover trials one may fail to detect an interaction even if present.

The current study used a single-blinded placebo controlled procedure and is therefore not completely free from bias. Nevertheless, placebo controlled studies are scarce if not exceptional in intracochlear electrical stimulation for tinnitus suppression while it is expected that the tinnitus population is highly sensitive to possible placebo effects [35]. Here, a minimal current level was applied to avoid warning signals on both the processor and remote control. This current level was sub-threshold and assumed to be unable to influence neural structures for tinnitus suppression. The current study needs to be interpreted with caution and future studies on the effectiveness of meaningless intracochlear electrical stimulation on tinnitus are highly recommended.

### Future perspectives

In the current study, coding of environmental sounds seems to be no requirement for tinnitus suppression using intracochlear electrical stimulation. Therefore, the relatively simple electrical stimulation, without a highly sophisticated speech processing strategy, might be a viable treatment option and could possibly reduce the production costs with respect to the standard clinical CI. A relatively simple pattern generator is sufficient for this treatment option. Moreover, it might be possible that the effectiveness of intracochlear electrical stimulation on tinnitus suppression could be further optimized using a combination of speech processing and the meaningless, but highly controlled, intracochlear electrical stimulation [36].

The results obtained from looped intracochlear electrical stimulation on tinnitus suppression could possibly be further optimized in future studies. Pre-operative ultra-high field (functional) magnetic resonance imaging of the central nervous system could for example show more accurate information about the optimal stimulation site inside the cochlea compared to the subjective tinnitus pitch matching procedure used in the current study. Furthermore, postoperative imaging using x-rays improves the knowledge about the position of the electrode array and prevent a possible mismatch between the assigned tinnitus pitch-matched electrode based on the default frequency allocation table in the standard clinical software [37] and the calculated tinnitus pitch-matched electrode based on the available frequency-position functions [38,39].

One might wonder what the profit is of replacing the perception of their own tinnitus by a sound perception induced by the intracochlear electrical stimulation. The sound perception induced by the electrical stimulation applied was experienced as more comfortable than their own tinnitus and often became inaudible over time due to loudness adaptation or a shift in attention [10]. The stimulus comfort was guaranteed by the subject-specific selection on a subjective base of the most convenient stimulation patterns. Moreover, subjects were allowed to reduce the current level of the looped stimulation during the crossover design in case of tinnitus reduction or stimulus discomfort.

Although the current study tested a great range of looped stimulation patterns, it is possible that the effectiveness and comfort could be optimized using other stimulation patterns. For example, although the optimal stimuli for tinnitus suppression appear to be subject-specific (preliminary work), we concluded that low amplitude electrical stimulation and high rate stimulation resulted in statistically more loudness adaptation compared to high amplitude electrical stimulation and low rate stimulation (unpublished data). Loudness adaptation improves stimulus comfort and therefore a combination of optimal tinnitus suppression and loudness adaptation may be preferred.

Furthermore, additional studies will give insight in the factors limiting the effectiveness of intracochlear electrical stimulation on tinnitus. In the current study it seems for example that stress hampered the effectiveness and there are possible indications that this proposed treatment option is suboptimal in case of a more central origin of the auditory deprivation. Future studies are needed for more evidence for these suggestions and in order to improve patient counselling and informed consent. It would also be interesting to investigate the effectiveness of looped intracochlear electrical stimulation in subjects with tinnitus complaints and significant residual hearing, in which a standard clinical CI would not be expected to have any audiological advantages.

### Conclusion

In the current placebo-controlled clinical trial a statistically significant tinnitus reduction was observed using intracochlear electrical stimulation. No statistically significant difference was found between looped intracochlear electrical stimulation (TI) and standard clinical CI. Furthermore, no significant effect of either CI or TI was found on depression and health-related quality of life. These results show that coding of environmental sounds does not appear to be a requirement for tinnitus suppression in the SSD population. Nevertheless, an adequate selection procedure seems to be essential for the effectiveness of the proposed treatment option. These results need to be interpreted with caution because of several methodological limitations and therefore future research is highly recommended.

## Supporting Information

S1 File CONSORT 2010 Checklist. (DOC)

S2 File Original Study Protocol. (PDF)
