# Supplementary material for: Tinnitus Suppression by Intracochlear Electrical Stimulation in Single Sided Deafness – A Prospective Clinical Trial: Follow-Up
Source: PLoS One. 2016 Apr 25;11(4):e0153131. doi: 10.1371/journal.pone.0153131 (PMC4844154; doi:10.1371/journal.pone.0153131)
Supplement: S2 File — (PDF) [file pone.0153131.s002.pdf]

RESEARCH PROTOCOL

Tinnitus suppression with electrical stimulation in subjects with Single Sided Deafness.

(Version 3.1 July 2014)

Drs. R.A.G.J. Arts

Prof. Dr. R.J. Stokroos

Academisch ziekenhuis Maastricht (azM)

PROTOCOL TITLE 'Tinnitus suppression with electrical stimulation generated by pattern generator using cochlear implants in single sided deafness patients.'

**Protocol ID**	**ABR-number: 38789**
**Short title**	**Tinnitus suppression with cochlear implants in subjects with Single Sided Deafness (SSD).**
**Version**	**3.1**
**Date**	**July 2014**
**Coordinating investigator/project leader/Principal investigator**	***Prof. Dr. R.J. Stokroos***<br>***Robert.stokroos@mumc.nl***<br>***043-3875590***
Researcher	***Drs. R.A.G.J. Arts***<br>***Remo.arts@mumc.nl***<br>***043-3872873***
**Sponsor**	***azM***<br>***P. Debyelaan 25***<br>***6229 HX Maastricht***<br>***Nederland***<br>***Tel: 043-3876543***
**Independent physician**	***Dr. K.W. Kross***<br>***Kenneth.kross@mumc.nl***<br>***043-3877585***
**Psychologist**	**Drs. A.E.C.C. Voss**<br>**+31649850789**
**Statistician**	***Dr. M. Chenault***<br>**043-3875597**<br>***Mickey.chenault@maastrichtuniversity.nl***
**Clinical physicist-Audiologist**	***Dr. E.L.J. George***<br>***+31-43-3874594***<br>**erwin.george@mumc.nl**

PROTOCOL SIGNATURE SHEET

Name	Signature	Date
**Sponsor or legal representative:**<br>***azM***<br>***Prof. Dr. R.J. Stokroos – ENT physician/ surgeon***		
**Coordinating Investigator/Project leader/Principal Investigator:**<br>***Prof. Dr. R.J. Stokroos – ENT physician/ surgeon***		

TABLE OF CONTENTS

1. INTRODUCTION AND RATIONALE 11
2. OBJECTIVES 12
3. STUDY DESIGN 13
4. STUDY POPULATION 14
   4.1 Population (base) 14
   4.2 Inclusion criteria 14
   4.3 Exclusion criteria 14
   4.4 Sample size calculation 14
5. TREATMENT OF SUBJECTS 15
   5.1 Investigational product/treatment 15
   5.2 Use of co-intervention (if applicable) 15
6. METHODS 23
   6.1 Study parameters/endpoints 23
      6.1.1 Main study parameter/endpoint 23
      6.1.2 Secondary study parameters/endpoints (if applicable) 23
      6.1.3 Other study parameters (if applicable) 23
   6.2 Randomisation, blinding and treatment allocation 23
   6.3 Study procedures 23
   6.4 Withdrawal of individual subjects 17
      6.4.1 Specific criteria for withdrawal (if applicable) 17
   6.5 Replacement of individual subjects after withdrawal 17
   6.6 Follow-up of subjects withdrawn from treatment 17
   6.7 Premature termination of the study 17
7. SAFETY REPORTING 18
   7.1 Section 10 WMO event 18
   7.2 Adverse and serious adverse events 18
      7.2.1 Suspected unexpected serious adverse reactions (SUSAR) 28
      7.2.2 Annual safety report 28
   7.3 Follow-up of adverse events 29
   7.4 Data Safety Monitoring Board (DSMB) 29
8. STATISTICAL ANALYSIS 20
   8.1 Descriptive statistics 20
   8.2 Univariate analysis 20
   8.3 Multivariate analysis 20
   8.4 Interim analysis (if applicable) 37
9. ETHICAL CONSIDERATIONS 38
   9.1 Regulation statement 39
   9.2 Recruitment and consent 39
   9.3 Objection by minors or incapacitated subjects (if applicable) 39
   9.4 Benefits and risks assessment, group relatedness 39
   9.5 Compensation for injury 39

9.6 Incentives (if applicable)....42
10. ADMINISTRATIVE ASPECTS AND PUBLICATION 42
10.1 Handling and storage of data and documents....43
10.2 Amendments....43
10.3 Annual progress report....43
10.4 End of study report....44
10.5 Public disclosure and publication policy 44
11. REFERENCES 44

LIST OF ABBREVIATIONS AND RELEVANT DEFINITIONS

ABR	ABR form, General Assessment and Registration form, is the application form that is required for submission to the accredited Ethics Committee (In Dutch, ABR = Algemene Beoordeling en Registratie)
AE	Adverse Event
AR	Adverse Reaction
BDI	Beck Depression Inventory
CA	Competent Authority
CCMO	Central Committee on Research Involving Human Subjects; in Dutch: Centrale Commissie Mensgebonden Onderzoek
CI	Cochlear Implant
CT	Computed Tomography
CV	Curriculum Vitae
dB	deciBel
DSMB	Data Safety Monitoring Board
EU	European Union
EudraCT	European drug regulatory affairs Clinical Trials
GCP	Good Clinical Practice
HL	Hearing Level
IB	Investigator’s Brochure
IC	Informed Consent
IMP	Investigational Medicinal Product
IMPD	Investigational Medicinal Product Dossier
METC	Medical research ethics committee (MREC); in Dutch: medisch ethische toetsing commissie (METC)
MRI	Magnetic Resonance Imaging
PTA	Pure Tone Average: average hearing threshold at 0.5, 1 and 2 kHz
PM	Tinnitus Pitch Matching
RI	Residual Inhibition
SADE	Serious Adverse Device Effect
(S)AE	(Serious) Adverse Event
SIN	Speech In Noise
SPC	Summary of Product Characteristics (in Dutch: officiële productinfomatie IB1-tekst)
SPL	Speech Pressure Level

**Sponsor**	**The sponsor is the party that commissions the organisation or performance of the research, for example a pharmaceutical company, academic hospital, scientific organisation or investigator. A party that provides funding for a study but does not commission it is not regarded as the sponsor, but referred to as a subsidising party.**
**SRT**	**Speech reception threshold**
**SSD**	**Single Sided Deafness**
**SUSAR**	**Suspected Unexpected Serious Adverse Reaction**
**THI**	**Tinnitus Handicap Inventory**
**TLM**	**Tinnitus Loudness Matching**
**TQ**	**Tinnitus Questionnaire**
**USADE**	**Unanticipated Serious Adverse Device Effect**
**VAS**	**Visual Analogue Scale**
**WBP**	**Personal Data Protection Act (in Dutch: Wet Bescherming Persoonsgevens)**
**WMO**	**Medical Research Involving Human Subjects Act (in Dutch: Wet Medisch-wetenschappelijk Onderzoek met Mensen**

SUMMARY

Rationale: Tinnitus (Aurium) is a symptom characterized by the perception of sound or noise in the absence of any objective external physical source. This disorder affects millions of people worldwide; its precise pathophysiologic mechanism is unknown. It has yet remained refractory to current medical treatment. It is supposed that tinnitus might be suppressed by restoring peripheral auditory neural activity. In clinical practice, conventional hearing aids are often used for this purpose with only limited success.

Scarce studies in a limited number of patients show that electrical stimulation of the (vestibulo)cochlear nerve proves beneficial in some cases[1-12]. The effect of peripheral electrical stimulation seems to depend on finding and applying the optimal stimulation pattern, as well as creating an optimal electroneural interface (i.e. the coupling of the electrode to the auditory nerve)[10,13].

We propose to stimulate the cochlear nerve by using cochlear implant (CI). A CI is a multi-channel device implanted in the cochlea which makes it possible to apply frequency- and amplitude specific stimuli to the cochlear nerve. It provides a relatively good electroneural interface and a lower surgical risk compared to direct stimulation of the auditory nerve.

To achieve speech understanding, the CI is programmable in various frequency bands. This feature enables us to program various electrical stimulation patterns to achieve tinnitus suppression. Thus, using CI for tinnitus suppression enables a more customised, patient-specific treatment, compared to direct neural stimulation. Moreover, applying a CI might also create possibilities to develop a tinnitus implant, enabling treatment for a debilitating condition for which currently no treatment is available.

In current CI-recipients who suffer from pre-implant tinnitus and who are profoundly deaf, generally a suppressive effect on tinnitus is reported in 65% to 93% of cases[2-12]. This suppressive effect may be caused by the electrical stimulation itself or by the shift in patient's attention from the tinnitus to environmental sounds[14].

Objective: The main objective of the study is to gain insight in the underlying mechanism of tinnitus suppression. This objective will be achieved by comparing the suppressive effect of a "simple electrical stimulation" pattern (i.e. without environmental sound perception) with the "standard stimulation pattern" of a CI (i.e. with environmental sound perception), in SSD-patients. A secondary objective is to investigate whether either of the two can be maintained over time.

Study design: There are indications that the optimum simple electrical stimulation pattern is subject specific. Therefore, a fine tune procedure will be performed to obtain the optimal stimulation pattern for each individual patient[15,16]. Subsequently, subjects will be assigned to one of two study groups. In group I, simple electrical stimulation will be followed by conventional CI usage, while in group II, conventional CI usage will be followed by simple electrical stimulation. After this crossover design the treatment modality of choice, either simple electrical stimulation or conventional CI, can be chosen by the patient. Outcomes will be analyzed between alternating conditions.

Study population: Ten Single Sided Deaf (SSD)-patients suffering from unilateral tinnitus will participate in this study. Strict in- and exclusion criteria will be applied, such that they can be expected to benefit from a CI in standard mode, in terms of localization and speech perception in noise.

Intervention (if applicable): Through a standard surgical procedure subjects will receive a CI in the ipsilateral deaf ear. After implantation and standard CI-fitting, a fine tune procedure will be performed to obtain the optimal stimulation pattern for each individual patient. Subsequently, the long term effect of either of both modes will be assessed in the crossover study. Finally, patients are able to choose their most comfortable treatment modality.

Main study parameters/endpoints: The main endpoint is the intensity of the experienced tinnitus measured with a Visual Analogue Scale. Secondary endpoints are two question-naires measuring tinnitus impact (Tinnitus Questionnaire and Tinnitus Handicap Inventory) and two objective audiological tests (Tinnitus Loudness Matching and Residual Inhibition).

Nature and extent of the burden and risks associated with participation, benefit and group relatedness: Participants will undergo audiometry and clinical imaging (MRI) as part of the routine diagnostics in single sided deafness. Cochlear implantation is a routine surgical procedure carried out about 80 times each year in the azM in children and adults, under general anaesthesia. The risk of major and minor complications associated with this surgical procedure is low in the azM compared to the literature[17]. Risks are wound infection, tempo-rary dysbalance, temporal ipsilateral facial nerve palsy and permanent loss of residual hearing in the affected ear, if any residual hearing is present.

All electrical stimulation patterns in the study will be applied using the standard CI-processor, remaining well within its conventional, clinical safety limits. Cochlear implantation involves highly patient-specific fitting, assuring that all stimulating patterns are optimized for each patient. The non-invasive audiological- and tinnitus-specific tests, plus various questionnaires at predetermined times take time and effort: ca 2 hours per visit, with a total of 20 visits.

Literature shows that cochlear implantation in SSD-patients provides a significant improvement in speech perception, specifically in spatial conditions, as well as in directional hearing and localization[18-21]. Also, the long term suppressive effect of the standard CI-processor (i.e. with environmental sounds perception) on tinnitus has been reported in certain cases and will possibly provide individual benefit for the included participants[22,23].

1. INTRODUCTION AND RATIONALE

Prevalence

Tinnitus (Aurium) is a symptom defined as a phantom sensation of sound in the absence of an external physical source. Tinnitus is affecting 10%-15% of the population with a high impact on general well being. About 3% of the population has severe tinnitus that leads to a significant decrease in quality of life[23]. Objective tinnitus is caused by objective internal sources. Subjective tinnitus arises without an objective internal source. Since 95% of patients suffer from subjective tinnitus, objective tinnitus is disregarded in this proposal.

Pathophysiology

The precise pathophysiologic mechanism underlying tinnitus has yet to be elucidated. Two hypothesis are generally accepted in literature:

I Tinnitus arises from a reduction in (or loss of) spontaneous neural activity in the deafferented regions of the cochlea, which in turn causes hyperactivity in the central auditory system by an imbalance of excitatory- or inhibitory neurotransmitter release[24].

II Tinnitus is caused by tonotopic reorganisation a result of a damaged hearing. This results in an increased synchrony in nerve firing in the auditory cortex and central auditory nuclei (such as the colliculus inferior) which may result in tinnitus[24].

Treatment

Tinnitus remains refractory to treatment: acoustic tinnitus masking, tinnitus retraining therapy and medical therapy are no more effective than placebo[25]. Tinnitus is possibly reversible by restoring peripheral auditory neural activity by means of electrical stimulation. Electrical stimulation can be applied directly to the auditory nerve or intracochlear.

Holm and colleagues reported in 4 of 5 tinnitus patients some benefit from direct electrical stimulation of the auditory nerve[1]. They expect their results to improve by optimizing the stimulation pattern used[10,13]. Direct stimulation of the auditory nerve is surgically challenging and poses a risk to the patient, associated with an intracranial intervention in the posterior cranial fossa.

Therefore, we propose to use electrical stimulation by intracochlear stimulation of the auditory nerve. A cochlear implant (CI) may be used for this purpose. Compared to direct stimulation of the auditory nerve, the electroneural interface is better and the surgical risk is lower in a cochlear implant compared to direct stimulation of the auditory nerve.

Cochlear implants (CI)

A CI is a multi-electrode device implanted in the cochlea which stimulates the cochlear nerve electrically. Each electrode is positioned in a specific area of the cochlea and is therefore

responsible for a specific pitch and loudness. It restores the ability to hear in a person who has a severe sensorineural hearing loss or who is deaf.

Literature shows that CI causes tinnitus suppression[6]. It appears that in deaf patients suffering from tinnitus, the experienced tinnitus level is reduced in 65% to 93% of cases[2-12]. Mechanisms responsible for this suppressed tinnitus effect could be:

- The shift in patient's attention from tinnitus to the environmental sounds, caused by improved hearing[14].
- Direct electrical stimulation; by artificially restoring the reduced (or lost) nerve activity i.e. to restore the code of silence.

The first mechanism probably explains why a majority of CI users experience tinnitus suppression when using their CI. From the second mechanism, it is apparent that it may be possible to suppress tinnitus without actually perceiving the electrical stimulus[10].

Simple electrical stimulation

The use of the CI in standard mode, including perception of environmental sounds, will be referred to as "standard CI". The use of electrical stimulation without perception of environmental sounds will be called "simple electrical stimulation". Simple electrical stimulation enables use of the unique ability of the CI to deliver "customised treatment" which is favourable since the optimal stimulus pattern to suppress tinnitus seem to be subject specific[10,13]. Simple electrical stimulation can be produced by a pattern generator, which is part of the standard CI processor.

The few studies on simple electrical stimulation are inconclusive on the optimal stimulation pattern:

Rubinstein et al. investigated tinnitus suppression with a simple electrical stimulation in a pilot study in bilaterally deaf subjects. A short term benefit was found in some subjects[25]. In this study, the long term effect was not investigated and only one possible stimulation pattern was examined. Furthermore, this study was hindered by the inability to pitch and loudness match the tinnitus using the contralateral ear since bilaterally deaf subjects were enrolled.

Recently, Zeng et al. published a case report of a patient with Single Sided Deafness (SSD), who received a CI in the deaf ear with tinnitus[15]. A simple electrical stimulation was generated by the CI. In this subject, the short-term tinnitus suppression with specific stimulation patterns proved beneficial. Some others have confirmed this observation[13,16,26,27]. No studies exist investigating the long term effect of simple electrical stimulation, or which investigate the ideal electrical stimulation pattern.

Single Sided Deafness (SSD)

In bilateral deaf subjects, one CI is reimbursed and implanted to restore environmental hearing. This makes them unsuitable to investigate simple electrical stimulation, since it abolishes the primary CI function and bereaves them of their hearing sensation. Secondly, bilaterally deafened subjects are unable to match tinnitus pitch and loudness, which seem to be important parameters in finding the optimal simple electrical stimulating pattern.

Therefore, it is proposed to examine the effect of simple electrical stimulation in SSD subjects. SSD-patients will still be able to hear environmental sounds and communicate using their contralateral, normal ear. Morever, SSD-patients are able to adequately match tinnitus pitch and loudness by making use of the healthy contralateral ear as a reference.

CI in SSD patients is currently not reimbursed by Dutch health authorities, although there is evidence in literature that CI is more suitable to re-establish binaural hearing. A CI in the deaf ear can be expected to largely restore directional hearing and to improve speech perception in noise [18-23]. Determining whether patients will additionally experience a suppressive effect on their tinitus is the main goal of the current study.

Literature shows two studies investigating the long term effect of standard CI in SSD-subjects. Van de Heyning et al. show that the standard CI-mode (i.e. with environmental sounds and speech perception) seems to have a suppressive effect on tinnitus also[22,23].

Moreover, Buechner et al. showed that when using the standard CI mode, SSD subjects benefit from improved speech perception and also appear to experience tinnitus suppression[19]. The objective of the present proposal is to understand the mechanism by which this electrical suppression of tinnitus operates.

2. OBJECTIVES

Generally, the purpose of this study is to investigate the effect of electrical stimulation on tinnitus in SSD-patients over time. The main objective is to gain insight in the underlying mechanism of tinnitus suppression. This objective will be achieved by comparing the suppressive effect of a “simple electrical stimulation” pattern (i.e. without environmental sound perception) with the “standard stimulation pattern” of a CI (i.e. with environmental sound perception). A secondary objective is to investigate whether either of the two can be maintained over time.

It is hypothesized that the suppressive effect of electrical stimulation by CIs on tinnitus is due to restoring the code of silence in the cochlea and is also feasible with a simple stimulation pattern (i.e. without environmental sound perception). Moreover, it is hypothesized that this effect can be maintained over time.

If it is indeed possible to reduce tinnitus by restoring the code of silence in a derived part of the auditory system by using a simple pattern generator, this would provide a treatment option for this debilitating condition. In the future, such a “tinnitus implant” might open new treatment perspectives to patients with a partial hearing deficit and severe tinnitus, to whom currently no treatment is available.

3. STUDY DESIGN

The study design is summarized in figure 1.

Figure 1: flow-chart

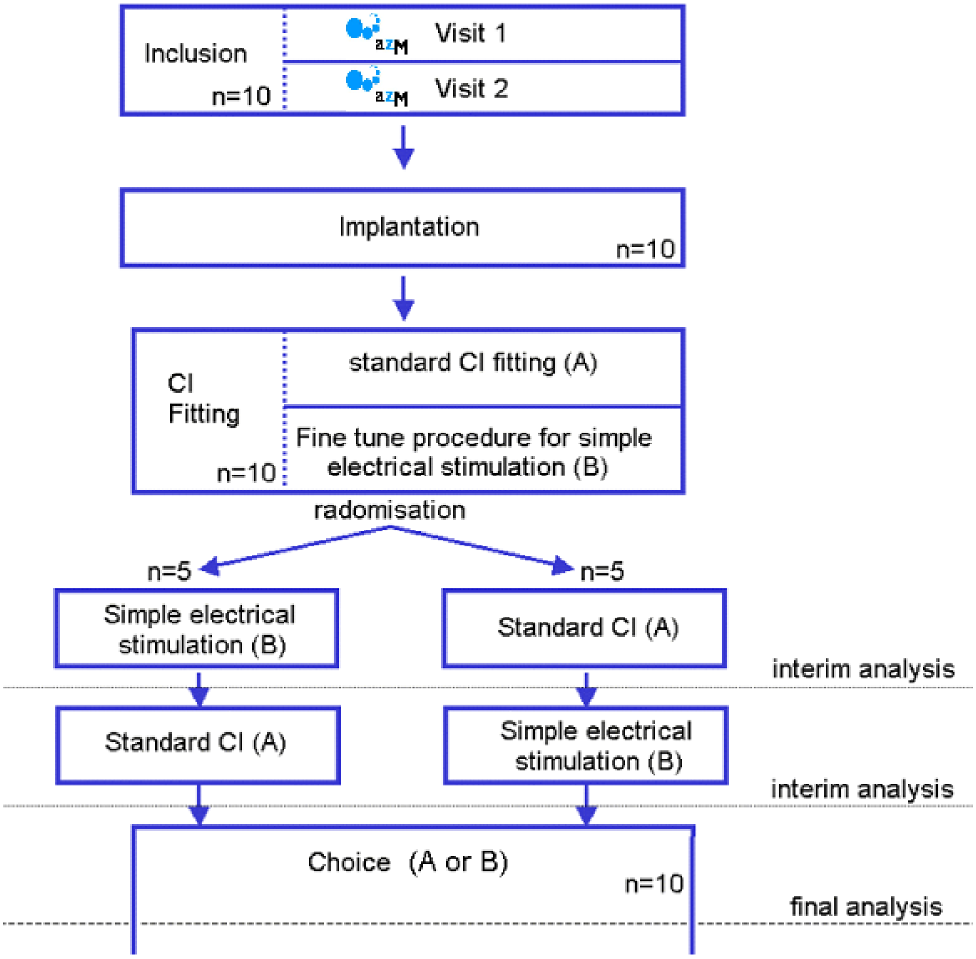

Implantation

Implantation will take place by means of mastoidectomy with posterior tympanotomy, round window approach (mpta). The device will surgically be implanted under a general anesthetic, and the operation usually takes from 1.5 to 5 hours. An incision will be made in the skin behind the ear and the surgeon drills into the mastoid bone, creating a pocket for the receiver/stimulator. Next, the electrode array will be inserted into the scala tympani of the cochlea through the round window. The patient normally goes home the same day or the day after the surgery, although some CI-recipients stay in the hospital for 1 day.

One week postoperatively the sutures will be removed. After 1-4 weeks of healing, the implant will be activated in the sixth week by connecting an external sound processor to the internal device via a magnet.

Standard CI fitting and fine tuning

After subject inclusion and CI implantation, the CI fitting procedure is started. First the fitting procedure for the implanted CI will be accomplished comparable to CI fitting in bilaterally deaf subjects in regular care. The CI-settings will be adjusted via the external part (processor) and are therefore painless. During this standardized fitting procedure subjects will get used to electrical stimulation of the auditory pathway in order to provide awareness of environmental sounds and speech understanding. The dynamic range* will be optimized. This procedure takes about three months and is called “standard CI fitting”.

Subsequently, the CI is deactivated for two weeks to wash out the possible influence on tinnitus of providing a hearing sensation. Then, simple electrical stimulation is applied to the CI in order to find the optimal simple electrical stimulation pattern that provides optimal tinnitus suppression. This procedure is called “fine tuning procedure for simple electrical stimulation”.

A fine tune procedure is necessary because the available literature indicates that the optimal simple electrical stimulation pattern is patient-specific[10]. This specific pattern will then be used during the crossover study. The fine tune procedure is conducted in four four-hour visits, within a two week period, to the azM. During these visits several stimulation patterns will be examined for six minutes each, scoring them on perceived tinnitus loudness and comfortability. Programming these stimulation patterns occurs via the external part (processor) and are therefore painless. Between these visits the CI will be deactivated to prevent it from affecting optimization results.

The following parameters are important in establishing the optimal simple electrical stimulation pattern: stimulus location (electrode), stimulation rate, amplitude (intensity) and modulation In detail, the test protocol consists of combinations of the following parameter settings:

Location:	-electrode responsible for the perceived tinnitus frequency
	-most basal located electrode
	-most apical located electrode
	-all 12 electrodes present
Rate:	-200 Hz (low)
	-750 Hz (mid)
	-6000 Hz (high)

* Dynamic range: the current levels between the inaudible current levels and current levels perceived as uncomfortable loud, as determined during the fitting procedure.

	-pitch matched (equal to the frequency of perceived tinnitus)
Amplitude:	-high current* (80% of dynamic range)
	-medium current (50% of dynamic range)
	-low current (20% of dynamic range)
Modulation:	-fixed amplitude (no modulation)
	-amplitude modulation

Crossover study

After establishing optimal parameter settings, a crossover study will be performed to compare the long term suppressive effects of standard CI (A) with the effect of the optimal simple stimulation pattern (B). Subjects will be randomly assigned to one of two study groups. In group I, simple electrical stimulation will be followed by conventional CI usage, while in group II, conventional CI usage will be followed by simple electrical stimulation. Modus (A) and (B) are switched after a three months interval since the effect on tinnitus reduction seems to stabilize after 3 months[23]. The total crossover will thus take 6 months (AB/BA); evaluation will take place after one and three months of using each mode. After 6 months, subjects are asked to choose their preferred condition (A or B) and a final analysis is made after another period of 3 months.

Table 1 reveals a schematic presentation of the measurements and a timepath.

* High current is expected to be perceived as uncomfortable (over time) and will therefore only be applied once, in combination with the pitch matched electrode and rate.

table 1: Overview of the study flow and planned measurements.

			Timepath (months) --->															
					0	1.5				2.5	3.5	4.5		5	5.5	6	12	15
			Selection		implantation	clinical fitting							deact.	fine tune procedure	deact.	crossover study	choise	end
measurements	Time (h)													total of **4 visits**		**5 visits**	**2 visits**	
**Audiological**	1	Audiometry		X						X	X	X		two times		each visit	each visit	
		Tympanometry		X						X	X	X		two times		each visit	each visit	
		Speech-In-Noise		X						X	X	X		two times		each visit	each visit	
**Tinnitus-specific**	0.25	Matching		X	X		X	X	X	X	X	X		each visit		each visit	each visit	
		RI														each visit	each visit	
		VAS	X		X		X	X	X	X	X	X		many times		each visit	each visit	
**Questionnaires**	0.25	TQ	X		X					X	X	X		two times		each visit	each visit	
		THI	X		X					X	X	X		two times		each visit	each visit	
		BDI	X													after 3 and 6 months	last visit	
		SSQ		X						X	X	X		two times		each visit	each visit	
		Tinnitus characteristics questionnaire	X		X					X	X	X		two times		each visit	each visit	
**Medical**	0.5	MRI		X														
		Subject's time invested (h)	0.25	1.75	0.5	1.25				1.5	1.5	1.5		15		9	3	
		**Total subject's time invested (h)**	**35.25**															

Table 1 shows a schematic overview of the study flow and planned measurements. The measurements are categorized in audiological, tinnitus-specific, questionnaires and medical. The 'X' represents the specified measurements in the timepath. For the *list of abbreviations*, see page 6. For explanation of the measurements, see *Study procedure*.

4. STUDY POPULATION

4.1 Population (base)

Participants are recruited from existing patient cohorts (ENT-department, azM and Adelante “Audiology and Communication”) and if necessary by advertising in patients associations (Nederlandse Vereniging Voor Slechthorenden, commissie tinnitus, and Tinnitus Vereniging Limburg). Subjects who are interested will first be informed about the study by mail by the investigator. After two weeks for reflection and after giving informed consent patients will be screened by filling in short questionnaires, followed by a phone-call by the investigator. When they are found to meet the criteria, they will be invited for a second visit to the azM for the medical check-up and audiological tests as part of the standard CI-selection *(see table 1).*

4.2 Inclusion criteria

- Ipsilateral: severely hearing-impaired (PTA more than 80 dB)
- Contralateral: moderate to normal hearing (PTA better than 50 dB)
  In cases of moderate hearing: PTA with hearing aid should be better than 20 dB.
- Experiencing tinnitus which is:
  - Continuous
  - Ipsilaterally localized
  - Characterized as a pure tone
  - Severe, that is:
    - Visual Analogue Scale-score > 7,
    - Tinnitus Handicap Inventory-score > 36 and/or Tinnitus Questionnaire-score > 41
  - Stable tinnitus (present > 2 years, stable > 1 year)
  - No reported suppressive effect from hearing aids
- Willingness to participate in this research (informed consent)

4.3 Exclusion criteria

- Pulsatile tinnitus
- History of psychiatric or neurological disorders or depression: Beck Depression Inventory score ≥ 20
- Use of antidepressant medication
- Cochleovestibular neurovascular conflict
- Congenital malformalities in auditory system
- History of vestibular schwannoma
- Ossified cochlea
- Active middle ear disease

- Age < 18 years old

4.4 Sample size calculation

Sample size calculation is based on data from a study by Van de Heyning and colleagues, who reported a statistically significant reduction in perceived tinnitus loudness with a standard CI in single sided deafness, measured on a visual analogue scale (1-10). After 24 months the mean was 2.5 (SD=1.9) compared to 8.5 (SD=1.3) before implantation[23].

Our study differs from this study in that we examine two interventions: standard speech processing and simple electrical stimulation. Therefore, in our study the standard speech processing will be considered the "control" intervention whereas simple electrical stimulation will be the experimental intervention. To compare the interventions with each other, the effect of both interventions relative to the situation before implantation will be determined.

For our calculation we take the s.d. of 1.9 rather than the pooled s.d. and consider a change in outcome of 2.5 as clinically relevant. An effect size of 1.86 is obtained:

The effect size (ES) of:

$$(\sqrt{2}*2.5/1.9=)\ 1.86$$

which is used to calculate the sample size, whereby further a power of 90% and an alpha-level of 0.05 for a two-sided paired-samples t-test (testing for difference in means) is considered. The calculation then results in a minimum of 8 subjects required.

A sample size of:

$$n=2(Z_\alpha + Z_\beta)^2/ES^2 = 2*(1.96 + 1.645)^2/ 1.86^2 = 7.52 \text{ rounded off this is 8.}$$

Allowing further for possible dropouts (approximately 20%), and to provide additional safety and efficacy information, it was decided to include a minimum of 10 subjects in the study. The funder (Med-El) agreed with the inclusion of 10 eligible subjects after independently performing sample size calculations.

5. TREATMENT OF SUBJECTS

5.1 Investigational product/treatment

For a schematic overview see *Flow-chart* and *table 1.*

Ten patients who meet the selection criteria will be implanted with a Med-El standard electrode SONATA ti100 using a standard surgical procedure performed by a CI-team with considerable experience in this procedure (> 500 cases). After this surgical procedure and recovery, the effect on tinnitus of the implantation itself, before activation of the CI is examined during an azM-visit necessary to remove sutures.

Next, a period of 3 months is planned to optimize the settings for the stimulation pattern of the speech processor, comparable to our department's standard fitting procedure after CI-implantation for deaf or severely hearing impaired subjects. Next, a period of two weeks is planned by which the CI is fully switched-off. This is necessary to wash out the possible influence on tinnitus by providing a hearing sensation.

Subsequently, the simple electrical stimulation pattern that best suppresses tinnitus and which is perceived as comfortable, will be determined in a fine tune procedure. The fine tune procedure consists of four visits, within a two week period, in which several stimulation patterns of 6 minutes each are applied. These stimulation patterns remain within or below the safe stimulation limits of commonly applied stimuli for auditory sensations. Examined parameters include location (electrode), rate, amplitude (intensity) and modulation. These parameters are explained in detail in the *study design.* The best pattern is established by the patient using a VAS scale in each stimulus applied. In this way "customised treatment" can be provided.

Subsequently, another period of one week is planned by which the CI is fully switched-off as an attempt to wash out the possible influence on tinnitus of providing simple electrical stimulation.

Finally, the patients participate in a randomized crossover study (AB/BA intervention study) for 6 months with a three months interval in which the patients at predetermined times visit the azM for audiological tests, tinnitus-specific tests and questionnaires. In this crossover study A stands for the standard CI function (control condition) and B for simple electrical stimulation. After these 6 months period another three months interval is started with the preferred condition (A or B). During this interval the same audiological tests, tinnitus-specific tests and questionnaires as during the crossover study will be performed at predetermined times.

5.2 Use of co-intervention

Because there is no cure for tinnitus yet, it is not likely that the patients will use any other intervention. If they do, however, they will be asked to stop this, at least for the duration of the current study.

6. METHODS

6.1 Study parameters/endpoints

6.1.1 Main study parameter/endpoint

The primary study variable is the intensity of the experienced tinnitus. The tinnitus intensity will be ranked on a Visual Analogue Scale (VAS). VAS is a quick measurement which is often used in comparable studies.

6.1.2 Secondary study parameters/endpoints (if applicable)

Because tinnitus is a subjective phenomenon, its intensity and impact are further identified by means of questionnaires (Tinnitus Questionnaire and Tinnitus Handicap Inventory) and audiological tests (Tinnitus Loudness Matching and Residual Inhibition) which have a more objective character. These secondary endpoints will give additional information which make the study outcome more robust.

6.1.3. Other study parameters (if applicable)

Because the intensity of the tinnitus may partly depend on the attention it is given to it by the patient, this study will also review the perceived quality of hearing. Based on a neurophysiologic model[14], a negative correlation may be expected between the quality of hearing and the intensity of the experienced tinnitus. This quality of hearing will be evaluated through non-invasive audiological tests (audiometry and Speech In Noise-test) and a questionnaire (Speech, Spatial and Qualities of hearing scale).

6.2 Randomisation, blinding and treatment allocation

Will be performed by computer randomisation using SPSS. The first six included subjects, in chronological order, will be assigned to one of two study groups. In group I, simple electrical stimulation will be followed by conventional CI usage, while in group II, conventional CI usage will be followed by simple electrical stimulation.

Finally, the last four included subjects, in chronological order, will also be divided into two equal groups of two subjects, group I or II.

We propose an open study (not blinded) since subjects will know which intervention is applied.

6.3 Study procedures

To determine whether the patient meets inclusion criteria and is suitable for CI implantation, several non-invasive standard clinical tests will be performed, which can be divided in four main categories:

Audiological tests
- Audiometry
- Tympanometry

Tinnitus-specific tests
- Visual Analogue Scales

Questionnaires
- Tinnitus Questionnaire
- Tinnitus Handicap Inventory
- Beck depression Inventory
- Speech, Spatial and Qualities of hearing scale
- Tinnitus characteristics questionnaire

Medical tests
- Magnetic Resonance Imaging

Participants will undergo audiometry and clinical imaging (Magnetic Resonance Imaging) as part of the routine diagnostics in Single Sided Deafness. Magnetic Resonance Imaging (MRI) is a medical imaging technique using magnetic resonance.

Patients who meet the in- and exclusion criteria will undergo Tinnitus Pitch- and Loudness Matching and additional audiological tests (see table 1; *Study design*).

On the day of surgery, a standard procedure for CI implantation in CI-candidates will be performed through a CI-team with considerable experience in this procedure.

One week after implantation the same non-invasive audiological tests, tinnitus-specific tests and questionnaires as before implantation will be used to characterize the tinnitus at predetermined times during the research (see table 1; *Study design*).

During the fine tune procedure for simple electrical stimulation, different stimulation patterns will be examined which are within or below the safe stimulation limits of commonly applied stimuli for auditory sensations (see *study design)*. After the optimal stimulation pattern has been found, a crossover design will be used to assess the long-term effect. After the crossover study subjects may choose their preferred intervention.

Measurements:

Audiological

- Audiometry (clinical standard): pure-tone- and speech audiometry

- Tympanometry (clinical standard): used to test the condition of the middle ear and mobility of the tympanic membrane and the conduction bones by creating variations of air pressure in the ear canal.
- SIN (Speech In Noise)-testing, to determine spatial speech intelligibility and auditory performance, using the Leuven Intelligibility Sentence Test (LIST)[28]. The LIST includes 35 lists of 10 sentences, spoken by a female speaker. In the adaptive test-procedure, the noise is presented at a constant level of 65 dB SPL, while the level of the speech signal is varied. Spatial configurations include both speech and noise presented from front (SoNo), speech presented from the front and noise from the CI side (SoNci), and noise presented from the front and speech presented from the CI side (SciNo)[18,28].

Tinnitus-specific

- PM (tinnitus Pitch Matching)[29]; using headphones, the pitch of the perceived tinnitus is estimated via a two-alternative forced-choice method. Measured at the contralateral, normal hearing ear.
- TLM (Tinnitus Loudness Matching)[29]; using headphones, the loudness of the perceived tinnitus is determined by presenting pure-tones at the contralateral, normal-hearing ear.
- RI (Residual Inhibition)[30]; the speech processor/pattern generator is deactivated and the amount of time is determined during which the patient still experiences a reduction of tinnitus loudness.
- VAS (Visual Analogue Scales; the left-hand side of the scale is assigned a score of 0, no tinnitus, very comfortable, no effect on daily activity or no problems due to tinnitus, and the right-hand side of the scale is assigned a score of 10, very loud disturbing tinnitus, very uncomfortable, extremely effect on daily activity or major problems due to tinnitus. The subjects have to mark with an X how they perceived the loudness, amount of discomfort, effect on life and extent of problems due to tinnitus, respectively)[31].

Questionnaires

- TQ (Tinnitus Questionnaire)[32]; consists of 52 questions, giving a description of common complaints seen in tinnitus patients. The degree of tinnitus is defined on the basis of the total score, maximum score: 84. A score of 0 to 30 is considered mild tinnitus degree, 31 to 46 corresponds to moderate tinnitus, severe tinnitus is a score from 47 to 59, and finally, a score of 60 or more corresponds to very severe tinnitus.
- THI (Tinnitus Handicap Inventory)[33]; an internationally validated evaluation score of the effects of tinnitus on patients emotions and daily activities. The THI consists of three subscales: a functional (12 items), an emotional (8 items) and a catastrophic

response subscale (5 items), and a total score which is the sum of these three outcomes. THI is a 25 self-administered questionnaire that aims to quantify the impact of tinnitus by measuring the perceived degree of handicap. Responders are asked to answer the questions with No (0 points), Sometimes (2 points) or Yes (4 points). The larger the total score is on the THI the more severe the handicap. A score of 0-16 is considered slight handicap, 18-36 corresponds to mild handicap, moderate handicap is a score from 38-56, 58-76 represents a severe handicap, and finally, a score of 78-100 corresponds to catastrophic handicap due to the tinnitus.

- BDI (Beck Depression Inventory)[34]; consists of 21 short questions, score 0-3, that especially stress the cognitive aspects of depression. A score of 0-13 represents a minimal depression, light depression is a score of 14-19, 20-28 corresponds to moderately serious depression, and finally, a score of 29 or more corresponds to serious depression.
- SSQ (Speech, Spatial and Qualities of hearing scale)[35]; comprises 49 items in three sections, the first section covering various aspects of hearing speech, the second on spatial hearing, and the third on a range of other qualities and features of hearing. Each item of the scale will be scored using a ruler, marked from 0-10, where 0 always represents minimal ability and 10 represents complete ability.
- Tinnitus characteristics questionnaire (a self-composed questionnaire for mapping characteristics as localization, timbre and constancy of the experienced tinnitus)

6.4 Withdrawal of individual subjects

If they wish to do so, subjects can leave the study, at any time for any reason and without any consequences. The investigator as well as the involved audiologist will guide the patients if desired. This is possible because of the relatively small study population (n=10). In addition, by choosing an intervention duration of three months we have tried to minimize the patient's burden since a prior study concluded that the suppressive effect on tinnitus is stabilized after 3 months[23]. Moreover, during the fine tune procedure for simple electrical stimulation, the stimulus-comfort is examined and only a comfortable stimulation pattern will be used during the crossover study.

The investigator and ENT physician / surgeon can decide to withdraw a subject from the study for urgent medical reasons. Therefore a drop-out of 20% is used in the sample size calculation.

6.4.1 Specific criteria for withdrawal

Can be any criterium which seriously affects the general (i.e. physical or mental) health status; withdrawal to be decided by investigator and the ENT physician.

6.5 Replacement of individual subjects after withdrawal

There will be no replacement of individual subjects after withdrawal.

6.6 Follow-up of subjects withdrawn from treatment

There will be no follow-up of subjects after withdrawal. One single visit to the azM is recommended to maintain CI settings for optimal use if desired.

6.7 Premature termination of the study

If premature termination of the study occurs, the cochlear implant will be converted to the standard speech perception mode (i.e. with environmental sounds and speech perception), to the pattern generator mode, or if desired, switched off completely.

7. SAFETY REPORTING

7.1 Section 10 WMO event

In accordance to section 10, subsection 1, of the WMO, the investigator will inform the subjects and the reviewing accredited METC if anything occurs, on the basis of which it appears that the disadvantages of participation may be significantly greater than was foreseen in the research proposal. The study will be suspended pending further review by the accredited METC, except insofar as suspension would jeopardise the subjects' health. The investigator will ensure that all subjects are continuously kept informed.

7.2 Adverse and serious adverse event/device effects

Adverse events are defined as any undesirable experience occurring to a subject during the study, whether or not considered related to the experimental treatment. All adverse events reported spontaneously by the subject or observed by the investigator or his staff will be recorded.

A Serious Adverse event (SAE) is any untoward medical occurrence or effect that:

- results in death;
- is life threatening (at the time of the event);
- requires hospitalisation or prolongation of existing inpatients' hospitalisation;
- results in persistent or significant disability or incapacity;
- is a congenital anomaly or birth defect;
- is a new event of the trial likely to affect the safety of the subjects, such as an unexpected outcome of an adverse event, major safety finding from a newly completed animal study, etc.

All SAEs and SADEs (see figure 2) will be reported through the web portal *ToetsingOnline* to the accredited METC that approved the protocol, within 15 days after the sponsor has first knowledge of the serious adverse reactions.

SAEs and SADEs that result in death or are life threatening should be reported expedited. The expedited reporting will occur not later than 7 days after the responsible investigator has first knowledge of the adverse event/effect. This is for a preliminary report with another 8 days for completion of the report.

7.2.1 Suspected unexpected serious adverse reactions (SUSAR)

In this study "Unanticipated Serious Adverse Device Effects" (USADE).

7.2.2 Annual safety report

7.3 Follow-up of adverse events

All adverse events will be followed until they have abated, or until a stable situation has been reached. Depending on the event, follow up may require additional tests or medical procedures as indicated, and/or referral to the general physician or a medical specialist.

7.4 Data Safety Monitoring Board (DSMB)

A Safety Monitoring Board (SMB) has been establishes to oversee the safety of the included subjects. The SMB consists of three independent members (members declared to have no competing interest):

-independent physician (not dr. K.W. Kross, since he is the independent physician of this study)

-statistician

-external clinical physicist/audiologist

This board will analyse “Serious Adverse (Device) effects” (SA(D)E’s) and is involved by reporting “Unanticipated Serious Adverse Device Effects” (USADE’s) and consequences of these USADE’s for the study. This board is also involved during interim analyses. The SMB performs an independent review of the collected safety reports from the study and makes recommendations to the PI and head of the department on further implementation of the study.

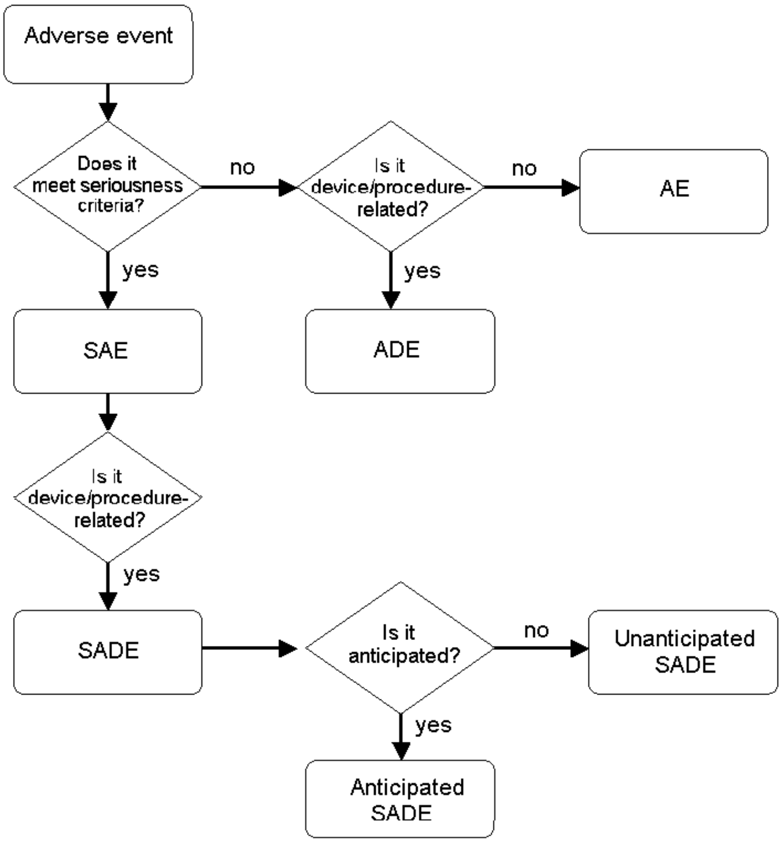

Figure 2: Adverse events categorization chart

Adverse Event (AE): Any untoward medical occurrence in a patient or clinical investigation subject administered a pharmaceutical product / device and which does not necessarily have a causal relationship with this treatment. An adverse event (AE) can therefore be any unfavourable and unintended sign (including an abnormal laboratory finding), symptom, or disease temporally associated with the use of a medicinal (investigational) product / device, whether or not related to the medicinal (investigational) product / device.

Serious Adverse Event: Any untoward medical occurrence that at any "dose":

- results in death,
- is life-threatening,
- requires inpatient hospitalization or prolongation of existing hospitalization,
- results in persistent or significant disability/incapacity, or
- is a congenital anomaly/birth defect

SMB-CHARTER

CONTENT	COMMENTS FROM TINNITUS STUDY
**1. Introduction**	
Name (and sponsor's ID) of trial plus ISRCTN and/or EUDRACT number	Tinnitus suppression with electrical stimulation in subjects with Single Sided Deafness. Sponsor's ID: METC 11-2-093 trial register: TC = 3374
Objectives of trial, including interventions being investigated	The main objective of the study is to gain insight in the underlying mechanism of tinnitus suppression. This objective will be achieved by comparing the suppressive effect of a "simple electrical stimulation" pattern (i.e. without environmental sound perception) with the "standard stimulation pattern" of a CI (i.e. with environmental sound perception), in SSD-patients. A secondary objective is to investigate whether either of the two can be maintained over time.
Outline of scope of charter	The purpose of this document is to describe the roles and responsibilities of the independent SMB for the METC 11-2-093 trial, including the timing of meetings, methods of providing information to and from the SMB, frequency and format of meetings, statistical issues and relationships with other committees.
**2. Roles and responsibilities**	
A broad statement of the aims of the committee	To protect and serve the included patients (especially re: safety) and to assist and advise Principal Investigators so as to protect the validity and credibility of the trial.<br>To safeguard the interests of trial participants and assess the safety and efficacy of the interventions during the trial.
Terms of reference	The SMB should receive and review the progress and accruing data of this trial and provide advice on the conduct of the trial to the Principal Investigator (PI).<br>The SMB should inform the PI if, in their view:<br>(i) the results are likely to convince a broad range of clinicians, including those supporting the trial and the general clinical community, that one trial arm is clearly indicated or contraindicated, and there was a reasonable expectation that this new evidence would materially influence patient management; **or**<br>(ii) it becomes evident that no clear outcome would be obtained."
Specific roles of SMB	Interim review of the trial's progress including updated figures on recruitment, data quality, and main outcomes and safety data.<br>A selection of specific aspects could be compiled from the following list:<br>• assess data quality, including completeness (and by so doing encourage collection of high quality data)<br>• monitoring evidence for treatment differences in the main efficacy outcome measures<br>• monitor evidence for treatment harm (eg USADEs, SAEs, deaths)<br>• decide whether to recommend that the trial continues to recruit participants or whether recruitment should be terminated either for everyone or for some treatment groups and/or some participant subgroups<br>• suggest additional data analyses<br>• advise on protocol modifications suggested by investigators or

CONTENT	COMMENTS FROM TINNITUS STUDY
	sponsors (eg to inclusion criteria, trial endpoints, or sample size)<br>• monitor compliance with previous SMB recommendations<br>• considering the ethical implications of any recommendations made by the SMB<br>• assess the impact and relevance of external evidence
**3. Before or early in the trial**	
Whether the SMB will have input into the protocol	All potential SMB members should have sight of the protocol/outline before agreeing to join the committee. Before recruitment begins the trial will have undergone review by a research ethics committee. Therefore, if a potential SMB member has major reservations about the trial (eg the protocol or the logistics) they should report these to the involved researcher and may decide not to accept the invitation to join. SMB members should be independent and constructively critical of the ongoing trial, but also supportive of aims and methods of the trial.
Whether the SMB will meet before the start of the trial	It is recommended that, if possible, the SMB meets before the trial starts or early in the course of the trial, to discuss the protocol, the trial, any analysis plan, future meetings, and to have the opportunity to clarify any aspects with the involved researchers (e.g. Principal Investigator). The SMB should meet within one year of recruitment commencing.<br>Consideration should be given to an initial “dummy” report, including the use of shell (empty) tables, to familiarise the SMB members with the format that will be used in the reports.
Any issues specific to the disease under study	Tinnitus can severely affect daily life and patients suffering from tinnitus must be accessed with due care.
Any specific regulatory issues	The SMB should be aware of any regulatory implications of their recommendations.
Whether members of the SMB will have a contract	Members of a SMB particularly for a commercially sponsored trial may be advised to have a contract making clear the need for confidentiality and the liability status of the SMB members. When there is no such contract, SMB members could formally register their assent by confirming (1) that they agree to be on the SMB and (2) that they agree with the contents of this Charter.
**4. Composition**	**1..1**
Membership and size of the SMB	Membership should consist of a small number of members, who include at least one clinician experienced in the clinical area and at least one statistician. Additional members experienced in clinical trials should reflect the other specialities involved in the trial. Consideration may be given to consumer representation, although they may be best represented on other committees. In the case of intergroup trials or trials with international collaboration consideration should be given to overseas members.<br>The members should be independent of the trial (eg should not be involved with the trial in any other way or have some competing interest that could impact on the trial). Any competing interests, both real and potential, should be declared. A short competing interest form should be completed and returned by the SMB members to the involved researcher (Annex 1).<br>The members of the SMB for this trial are:<br>(1) *Ir. Drs. A. Kessels (independent statistician)*<br>(2) *Ir. D.J.W.M. Scheijen (independent clinical physicist in audiology)*

CONTENT	COMMENTS FROM TINNITUS STUDY
	(3) Drs. J.W. Brunings (independent ENT-physician)
The Chair, how they are chosen and the Chair's role.	The Chair should have experience of chairing meetings, and should be able to facilitate and summarise discussions. The Chair is chosen by the SMB members themselves. The Chair is expected to facilitate and summarise discussions.
The responsibilities of the SMB statistician	The SMB membership will include a statistician to provide independent statistical expertise.
The responsibilities of the trial statistician	The trial statistician, *Dr. M. Chenault* will produce (or oversee the production of) the report to the SMB and will participate in SMB meetings, guiding the SMB through the report, participating in SMB discussions and, on some occasions, taking notes.
The responsibilities of the PI and other members of the Trial Management Group (TMG)	The PI, may be asked, and should be available, to attend open sessions of the SMB meeting. The other TMG members will not usually be expected to attend but can attend open sessions when necessary (See Organisation of SMB Meetings).
**5. Relationships**	
Clarification of whether the SMB are advisory (make recommendations) or executive (make decisions)	It is customary that the SMB does not make decisions about the trial, but rather makes recommendations to an appropriate executive committee or the PI.
The need for SMB members to disclose information about any competing interests	Competing interests should be disclosed. These are not restricted to financial matters – involvement in other trials or intellectual investment could be relevant. Although members may well be able to act objectively despite such connections, complete disclosure enhances credibility. (See Annex 1)<br>SMB members should not use interim results to inform trading in "medical device-company" shares, and careful consideration should be given to trading in stock of companies with competing products.
**6. Organisation of SMB meetings**	
Expected frequency of SMB meetings	The exact frequency of meetings will depend upon any statistical plans specified, and otherwise on trial events. The wishes of the SMB and needs of the trial office will be considered when planning each meeting. It is recommended that the SMB meet at least yearly.
Whether meetings will be face-to-face or by teleconference	The first meeting should ideally be face-to-face to facilitate full discussion and allow members to get to know each other. It is recommended that all subsequent meetings should be face-to-face if possible, with teleconference as a second option.
How SMB meetings will be organised, especially regarding open and closed sessions, including who will be present in each session	A mixture of open and closed sessions is recommended. Closed and open sessions should be defined. Commonly, only SMB members and others whom they specifically invite, eg the trial statistician, are present in closed sessions. In open sessions,_all those attending the closed session are joined by the PI(s), and sometimes also by the involved audiologist or researcher, as relevant.<br><br>The format of the meetings should be described.<br>1. Open session: Introduction and any "open" parts of the report<br>2. Closed session: SMB discussion of "closed" parts of the report and, if necessary,<br>3. Open session: Discussion with other attendees on any matters arising from the previous session(s).

CONTENT	COMMENTS FROM TINNITUS STUDY
	4. Closed session: extra closed session
**7. Trial documentation and procedures to ensure confidentiality and proper communication**	
Intended content of material to be available in open sessions	Open sessions: Accumulating information relating to recruitment and data quality (eg data return rates, treatment compliance) will be presented. Harmfulness details based on pooled data will be presented and total numbers of events for the primary outcome measure and other outcome measures may be presented, at the discretion of the SMB.
Intended content of material to be available in closed sessions	Closed sessions: In addition to all the material available in the open session, the closed session material will include efficacy and safety data by treatment group.
Will the SMB be blinded to the treatment allocation	Blinding is generally not recommended for SMB members, although opinions vary.
Who will see the accumulating data and interim analysis	The people who will see the accumulating data and interim analysis should be specified. The PI and head of the department will receive these data and interim analysis.<br><br>SMB members do **not** have the right to share confidential information with anyone outside the SMB, excluding the PI and involved audiologist and researcher.
Who will be responsible for identifying and circulating external evidence (eg from other trials/ systematic reviews)	Identification and circulation of external evidence (eg from other trials/ systematic reviews) is not the responsibility of the SMB members. The PI, involved researcher or the trials office team will usually collate any such information.
To whom the SMB will communicate the decisions/ recommendations that are reached	The SMB usually reports its recommendations in writing to the PI or sponsor's representative. This should be copied to the trial statistician (or trial manager) and if possible should be sent via the trials office in time for consideration at a trial steering committee (TSC) meeting. If the trial is to continue largely unchanged then it is often useful for the report from the SMB to include a summary paragraph suitable for trial promotion purposes. (See Annex 2.)
Whether reports to the SMB be available before the meeting or only at/during the meeting	It is usually helpful for the SMB to receive the report at least 2 weeks before any meetings. Depending on the trial, it may sometimes be preferable for all papers to be brought to face-to-face meetings by the trial statistician; time would then be needed for SMB members to assimilate the report.
What will happen to the confidential papers after the meeting	The SMB members should store the papers safely after each meeting so they may check the next report against them. After the trial is reported, the SMB members should destroy all interim reports.
**8. Decision making**	
What decisions/recommendations will be open to the SMB	Possible recommendations could include:<br>• No action needed, trial continues as planned<br>• Early stopping due, for example, to clear benefit or harm of a treatment, futility, or external evidence<br>• Stopping recruitment within a subgroup<br>• Extending recruitment (based on actual control arm response

CONTENT	COMMENTS FROM TINNITUS STUDY
	rates being different to predicted rather than on emerging differences) or extending follow-up<br>• Stopping a single arm of a multi-arm trial<br>• Sanctioning and/or proposing protocol changes
The role of formal statistical methods, specifically which methods will be used and whether they will be used as guidelines or rules	This Charter should include or provide reference to the planned interim analyses and statistical guidelines, ie the SMB should review and agree any interim analysis plan.<br>Formal statistical methods are more generally used as guidelines rather than absolute rules. This is because they generally only consider one dimension of the trial. Reasons should be recorded for disregarding a stopping guideline.
How decisions or recommendations will be reached within the SMB	Issues to be specified can include:<br>• The decision making methods and criteria that will be adopted for guiding deliberations<br>• The process of decision making, including whether there will be voting or other formal methods of achieving consensus. The method of deliberation should not be revealed to the overseeing committee as this may reveal information about the status of the trial's data.<br>• The role of the Chair - to summarise discussions and encourage consensus; it may be best for the Chair to give their own opinion last.<br>It is recommended that every effort should be made for the SMB to reach a unanimous decision. If the SMB cannot achieve this, a vote may be taken, although details of the vote should not be routinely included in the report to the PI and head of the department as these may inappropriately convey information about the state of the trial data.<br>It is important that the implications (eg ethical, statisticial, practical, financial) for the trial be considered before any recommendation is made.
When the SMB is quorate for decision-making	There should be a minimum number of attendees before the SMB is quorate for decision-making; this should be specified.<br>Effort should be made for all members to attend. The trials office team will try to ensure that a date is chosen to enable this. Members who cannot attend in person should be encouraged to attend by teleconference. If, at short notice, any SMB members cannot attend at all then the SMB may still meet if at least one statistician and one clinician, including the Chair (unless otherwise agreed), will be present. If the SMB is considering recommending major action after such a meeting the SMB Chair should talk with the absent members as soon after the meeting as possible to check they agree. If they do not, a further teleconference should be arranged with the full SMB.
Can SMB members who cannot attend the meeting input	If the report is circulated before the meeting, SMB members who will not be able to attend the meeting may pass comments to the SMB Chair for consideration during the discussions.
What happens to members who do not attend meetings	If a member does not attend a meeting, it should be ensured that the member is available for the next meeting. If a member does not attend a second meeting, they should be asked if they wish to remain part of the SMB. If a member does not attend a third meeting, they should be replaced.

CONTENT	COMMENTS FROM TINNITUS STUDY
Whether different weight will be given to different endpoints (eg safety/efficacy)	Safety is most important.
**9. Reporting**	
To whom will the SMB report their recommendations/decisions, and in what form	Usually, this will be a letter to the PI and head of the department. A timescale should be specified eg usually within 3 weeks. It is helpful if a copy of this is lodged with the trial office.
Whether minutes of the meeting be made and, if so, by whom and where they will be kept	Minutes will only be made in open sessions. They are made by a SMB member and stored by the SMB Chair. The SMB Chair should sign off any minutes or notes.
What will be done if there is disagreement between the SMB and the body to which it reports	If the SMB has serious problems or concerns with the TSC decision a meeting of these groups should be held. The information to be shown would depend upon the action proposed and the SMB's concerns. Depending on the reason for the disagreement confidential data will often have to be revealed to all those attending such a meeting. The meeting should be chaired by a senior member of the trials office staff or an external expert who is not directly involved with the trial.
**10. After the trial**	
Publication of results	At the end of the trial there may be a meeting to allow the SMB to discuss the final data with principal trial investigators/sponsors and give advice about data interpretation<br>The SMB may wish to see a statement that the trial results will be published in a correct and timely manner.
The information about the SMB that will be included in published trial reports	SMB members should be named and their affiliations listed in the main report, unless they explicitly request otherwise. A brief summary of the timings and conclusions of SMB meetings should be included in the body of this paper.
Any constraints on SMB members divulging information about their deliberations after the trial has been published	It should be specific when the SMB may discuss issues from their involvement in the trial eg 12 months after the primary trial results have been published, or when permission is agreed with the overseeing committee.

8. STATISTICAL ANALYSIS

8.1 Descriptive statistics

Because tinnitus is a highly subjective phenomenon, any quantification of tinnitus is subjective.

The descriptive statistics presented will include mean and SD, median and interquartile range, and maximum and minimum values. Qualitative data will be presented as absolute and relative frequencies.

This study with its limited number of subjects (10 subjects) may preclude applying parametric statistical testing. Normality will be assessed by examining histograms and performing the Kolmogorov-Smirnov test.

Depending on whether normality can be established, either nonparametric or parametric statistical tests will be performed. There are two measurements in time with two different orders of experimental condition, namely A-B and B-A where A is the standard CI and B is the simple electrical stimulation generated by the pattern generator. Thus either repeated measures ANOVA or the Friedman test will be performed. Also, paired sample t-testing or the Wilcoxon rank test will be performed for the two experimental conditions separately to examine any change in time. Furthermore, Spearman correlation coefficients will be obtained to compare the Tinnitus Handicap Inventory and the VAS scores. Furthermore, graphical presentations will be used to illustrate findings and compare the various outcomes.

8.2 Univariate analysis

It is expected that non-parametric tests such as the Wilcoxon rank sum test for pair-wise comparisons of scores per person and the Friedman test for comparing multiple scores per person will be applied. These values will be compared to the initial tinnitus scores.

8.3 Multivariate analysis

Spearman correlation coefficients will be obtained to compare the Tinnitus Handicap Inventory and the VAS scores. The spearman correlation will also be used to compare the quality of speech perception with the VAS scores. Furthermore, graphical presentations will be used to illustrate findings. The relation between these scores will be examined with adjustment for age, audiometric value, and also for initial tinnitus scores.

8.4 Interim analysis

It is assumed that withdrawal will occur if the subject thinks the experimental treatment is unpleasant.

If unexpected damage occurs, the researcher (in consultation with the ENT-physician) may choose not to continue with this subject. A 20% dropout is expected.

Interim analyses are established in the protocol to assess possible problems with the intervention or whether one condition appears to be very superior to the other. For these analyses, Beck Depression Inventory scores and tinnitus specific measurements will be evaluated.

Interim analyses shall occur after 3 and 6 months, during the crossover study for both groups to assess patient’s burden of the intervention. The seventh to the tenth patient (in chronological order) will only receive a CI when the interim analysis reveals no major burden during the crossover study or, based on this data, no major disadvantage from the intervention is expected. Figure 2 shows a schematic presentation of the long-term crossover design.

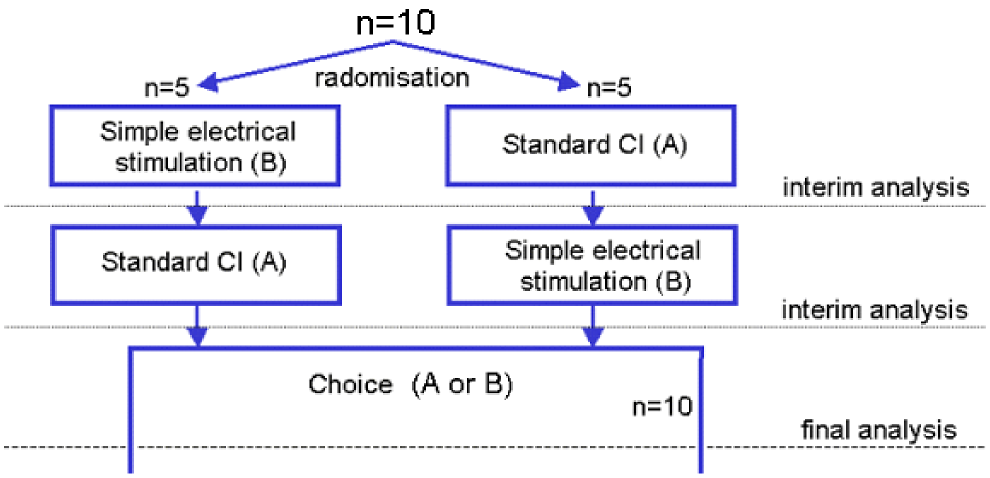

Figure 2: Schematic presentation of the long-term, crossover design. Subjects will follow either AB or BA depending on randomization. A represents the standard CI and B represents the simple electrical stimulation generated by the pattern generator. Three months/interval will be used. After this study subjects may continue the preferred intervention (A or B) for another three months period before the final analysis is made.

9. ETHICAL CONSIDERATIONS

9.1 Regulation statement

The study will be conducted according to the principles of the Declaration of Helsinki, sixth revision (2008), and in accordance with the Medical Research Involving Human Subjects Act (WMO).

9.2 Recruitment and consent

Patients will be acquired through recruitment from existing patient cohorts (ENT-department azM and Adelante "Audiology and Communication") by audiologists and ENT-specialists and if necessary via advertising in patients associations (Nederlandse Vereniging Voor Slechthorenden, commissie tinnitus and Tinnitus Vereniging Limburg). Preliminary consultations will take place after informed consent is given. Prior to the informed consent the study information is given. Study information is sent to patients who initially seem to be eligible. The researcher is responsible for an oral discussion, to be sure that the study information is understood, and the informed consent procedure. After informed consent is given, the researcher (in cooperation with an ENT surgeon and audiologist) will select eligible patients on the basis of questionnaires. The questionnaires, consisting of the tinnitus characteristics questionnaire, THI, TQ, BDI and four VAS-scales, will be filled in during an azM visit as part of the first selection procedure. The researcher will contact the possible subjects for their decision again to give them the ability to revise their decision. The researcher invite them for further selection (medical and audiologic) at our ENT-department (for more detail, see *table 1* and *inclusion and exclusion criteria*). For these potential subjects it is possible to contact the researcher if there are still questions. If desired, it is also possible to contact the independent physician, the ENT-surgeon or the involved audiologist.

9.3 Objection by minors or incapacitated subjects

Not applicable

9.4 Benefits and risks assessment, group relatedness

Participants will undergo a standard surgical procedure for CI implantation in their deaf ear as part of regular care for CI-candidates. This will be performed by a cochlear implant team with considerable experience in this procedure. The risk of major and minor complications associated with this surgical procedure is low, and in the Maastricht University Medical Centre (MUMC+) slightly lower than in literature[17].

Specifically, a potential risk (0.5/100) of CI surgery is the possible damage to the facial nerve, leading to reduced facial movement on one side. To reduce this risk, a facial nerve-monitor is used during surgery. If this complication does occur, it is in general temporarily. Another rare complication is wound infection. To prevent this the patient will be treated with antibiotic profylaxis before and after surgery. Occasionally, temporary changes in taste, a reduced sense of balance and a reduced sensation around the surgical side could be observed. The risks related to participating this study will be made clear in detail in the patient information which will be sent by mail and will be discussed on the first azM-visit.

During the study, subjects with mental complaints can contact the involved psychologist or the psychologist can contact them.

Table 2 shows a risk analysis in which the risks associated by participation are involved.

Table 2: Risk analyses.

**Risk**	**chance**	**Consequence**	**What will be done to reduce risk**
Facial nerve damage (n.7) during surgery	0.5%	Reduced facial movement on one side.	Use of nerve monitor during surgery.
Contamination of surgical side	rare	Infection of surgical side (wound infection).	Before as well as after surgery treatment with antibiotic profylaxis.
	Occasionally	Temporary changes in taste, reduced sense of balance and a reduced sensation around the surgical side.	
Too few results caused by premature termination of patients.		Possible conclusions not substantiated by statistics.	-proper patient information<br>-20% drop-out used<br>-inclusion of patients without psychiatric problems.
Psychological damage to patients.		Suicide or other complications	-proper patient information<br>-coaching if desired<br>-BDI: 20 or more is exclusion criterion.
(Serious) Adverse Device effects	Unknown	Damage to patient’s health	Stimulation parameters remains within or below the safe stimulation limits

			of commonly applied stimuli for auditory sensation
Meningitis	Sporadic case report. Never happened in the Netherlands, occasional	Development of meningitis after implantation.	All CI-clinics in the Netherlands advise vaccination against meningitis for all CI-candidates
Damage ipsilateral auditory organ (cochlea)	0-30%	The disappearance of any remaining natural hearing at implanted side, if any residual hearing is present.	Inclusion of subjects without usable hearing

After completion of the investigation, the included patients may keep the implanted CI without any additional costs, giving them an expected individual benefit, in cases of standard CI use, in directional hearing, spatial listening, speech perception in noise and possibly reducing the impact of tinnitus on their daily life[1,18-22]. They will receive a CI for an indication which health care insurance companies will not reimburse in the Netherlands. The CI settings used to restore hearing is one of the conditions used in our study (control). Otherwise, if they prefer, subjects are allowed to use simple electrical stimulation after study termination.

If the present study shows a significant long-term tinnitus suppression by stimulating the auditory nerve with a simple electrical pattern, a cure for this debilitating condition which affects 10%-15% of the population, and for which no cure is present yet, is found. In that case, the present study will initiate the development of a simple, low cost, tinnitus implant which suppresses tinnitus significantly by restoring the code of silence. This device allows customised health care for tinnitus which the standard CI mode does not allow.

In summary, considering the severity of the condition of tinnitus, the high impact tinnitus may have on daily life, the high prevalence, the fact that there is no medical cure and the possible scientific study outcome, weighted against the necessary investment of patient's time and effort and the limited risks, the investigator thinks this study is justified.

Group relatedness

It is proposed to examine the effect of simple electrical stimulation in SSD subjects. SSD-patients will still be able to hear environmental sounds and communicate using their contralateral, normal ear. Morever, SSD-patients are able to adequately match tinnitus pitch and loudness by making use of the healthy contralateral ear as a reference.

9.5 Compensation for injury

The sponsor/investigator has a liability insurance which is in accordance with article 7, subsection 6 of the WMO.

The sponsor (also) has an insurance which is in accordance with the legal requirements in the Netherlands (Article 7 WMO and the Measure regarding Compulsory Insurance for Clinical Research in Humans of 23th June 2003). This insurance provides cover for damage to research subjects through injury or death caused by the study.

1. € 450.000,-- (i.e. four hundred and fifty thousand Euro) for death or injury for each subject who participates in the Research;
2. € 3.500.000,-- (i.e. three million five hundred thousand Euro) for death or injury for all subjects who participate in the Research;
3. € 5.000.000,-- (i.e. five million Euro) for the total damage incurred by the organisation for all damage disclosed by scientific research for the Sponsor as ‘verrichter’ in the meaning of said Act in each year of insurance coverage.

The insurance applies to the damage that becomes apparent during the study or within 4 years after the end of the study.

9.6 Incentives

The SSD-patients included in this study will be kept free of any charges for the device, the implantation and any adjustments of the CI-settings. They will receive a cochlear implant for an indication which health care insurance companies will not reimburse in the Netherlands. Cochlear implants are only reimbursed in case of bilateral severe to profound sensorineural hearing loss. During our study and after study termination, we expect that the subject will benefit from the CI with regard to speech perception in noise, directional hearing and reduced tinnitus intensity[18,19].

10. ADMINISTRATIVE ASPECTS AND PUBLICATION

10.1 Handling and storage of data and documents

Data will be encrypted with a coded and stored. The key to the code is safeguarded in a file which is only accessible for the individuals who participate in this research project. Data will not be stored longer than necessary. The Academic Hospital Maastricht (azM), where this project will take place, maintains strict requirements for ensuring the privacy of patients.

Data collected during study can be classified in ‘clinical data’ or ‘study results’. Clinical data consists of information for the medical file which will be saved as a regular medical file. This is necessary for the aftercare. The study results will be stored for 15 years after study termination which is in agreement with the “wet bescherming persoonsgegevens” and the “International Conference on Harmonisation (ICH)/WHO Good Clinical Practice (ICH GCP)”[36].

10.2 Amendments

Amendments are changes made to the research after a favourable opinion by the accredited METC has been given. All amendments will be notified to the METC that gave a favourable opinion.

A ‘substantial amendment’ is defined as an amendment to the terms of the METC application, or to the protocol or any other supporting documentation, that is likely to affect to a significant degree:

- the safety or physical or mental integrity of the subjects of the trial;
- the scientific value of the trial;
- the conduct or management of the trial; or
- the quality or safety of any intervention used in the trial.

All substantial amendments will be notified to the METC and to the competent authority.

Non-substantial amendments will not be notified to the accredited METC and the competent authority, but will be recorded and filed by the sponsor.

Examples of non-substantial amendments are typing errors and administrative changes like changes in names, telephone numbers and other contact details of involved persons mentioned in the submitted study documentation.

10.3 Annual progress report

The sponsor/investigator will submit a summary of the progress of the trial to the accredited METC once a year. Information will be provided on the date of inclusion of the first subject, numbers of subjects included and numbers of subjects that have completed the trial, serious adverse events/ serious adverse reactions, other problems, and amendments.

10.4 End of study report

The investigator will notify the accredited METC of the end of the study within a period of 8 weeks. The end of the study is defined as the last patient's last visit.
In case the study is ended prematurely, the investigator will notify the accredited METC, including the reasons for the premature termination.
Within one year after the end of the study, the investigator/sponsor will submit a final study report with the results of the study, including any publications/abstracts of the study, to the accredited METC.

10.5 Public disclosure and publication policy

Sponsor (AzM) and Financier (MEDEL inc.) acknowledge the importance of public disclosure/publication of information collected or generated by the researchers or Principal Investigator engaged in this research. All results from the research project (whether positive or negative; expected or unexpected) will be publicly disclosed by Sponsor or Researcher at symposia, professional meetings, or in journals, theses or otherwise, in reasonable scientific cooperation with the financier, as far as disclosure is not in strict conflict with possible pending patents. So, the financier does not have any influence on publication of results, however, the financier will receive the article sixty (60) days prior to public disclosure to enable protection of confidential information or for possible patents.

11. REFERENCES

[1] Holm AF, Staal MJ, Mooij JJA, Albers FWJ. Neurostimulation as a new treatment for severe tinnitus: A pilot study. Otology & Neurotology 2005;26:425-428

[2] Di Nardo W, Cantore I, Cianfrone F, Melillo P, Scorpecci A, Paludetti G. Tinnitus modifications after cochlear implantation. Eur Arch Otorhinolaryngol 2007; 264:1145-1149

[3] Miyamoto R.T, Bichey B.G. Cochlear implantation for tinnitus suppression. Otolaryngol Clin N Am 2003; 36:345-352

[4] Bovo R, Ciorba A, Martini A. Tinnitus and cochlear implants. Auris Nasus Larynx 2010;xx:xxx-xxx

[5] Chadha NK, Gordon KA, James AL, Papsin BC. Tinnitus is prevalent in children with cochlear implants. International journal of Pediatric Otorhinolaryngology 2009;73:671-675

[6] Demajumdar R, Stoddart R, Donaldson I, Proops DW. Tinnitus, cochlear implants and how they affect patients. The Journal of Laryngology and Otology 1999;113:24-26

[7] Aschendorff A, Pabst G, Klenzner T, Laszig R. Tinnitus in cochlear implant users: The Freiburg experience. International Tinnitus Journal 1998;Vol.4,No2:162-164

[8] Ruckenstein MJ, Hedgepeth C, Rafter KO, Montes ML, Bigelow DC. Tinnitus suppression in patients with cochlear implants. Otology & Neurotology 2001;22:200-204

[9] Amoodi HA, Mick PT, Shipp DB, Friesen LM, Nedzelski JM, Chen JM, Lin VYW. The effects of unilateral cochlear implantation on the tinnitus handicap inventory and the influence on quality of life. The Laryngoscope 2011;121:1536-1540

[10] Tyler RS, Rubinstein J, Pan T, Chang S, Gogel SA, Gehringer A, Coelho C. Electrical stimulation of the cochlea to reduce tinnitus. Semin Hear. 2008;29(4):326-332

[11] Ito J, Sakakihara J. Suppression of tinnitus by cochlear implantation. American Journal of Otolaryngology 1994;Vol 15, No 2:145-148

[12] Palau EM, Gil JLM, Vidal CM, González JCF, Cabrera OA, Macías ÄR. Tinnitus and cochlear implantation. Preliminary experience. Acta Otorrinolaringol Esp. 2010;61(6):405-411

[13] Reavis KM, Chang JE, Zeng FG. Patterned sound therapy for the treatment of tinnitus. The hearing journal 2010; Vol.63 No.11:21-24

[14] Parazzini M, Del Bo L, Jastreboff M, Tognola G, Ravazzani P. Open ear hearing aids in tinnitus therapy: An efficacy comparison with sound generators. International Journal of Audiology 2011; 50:548-553

[15] Zeng F-G, Tang Q, Dimitrijevic A, Starr A, Larky J, Blevins N.H. tinnitus suppression by low-rate electric stimulation and its electrophysiological mechanisms.Hearing Research 2011;277:61-66

[16] Chang E, Fine E.L, Rothholtz V.S, Djalilian H, Zeng F-G. Variability in tinnitus suppression via electric stimulation. Hearing & Speech lab 2010; Poster presentation

[17] Postelmans JTF, Cleffken B, Stokroos RJ. Post-operative complications of cochlear implantation in adult and children: five years' experience in Maastricht. The Journal of Laryngology & Otology 2007; 121:318-323

[18] Vermeire K, Van de Heyning P. Binaural hearing after cochlear implantation in subjects with unilateral sensorineural deafness and tinnitus. Audiol Neurotol 2009;14:163-171

[19] Buechner A, Brendel M, Lesinski-Schiedal A, Wenzel G, Frohne-Buechner C, Jaeger B, Lenarz T. Cochlear implantation in unilateral deaf subjects associated with ipsilateral tinnitus. Otology & Neurotology 2010;311 1381-1385

[20] Arndt S, Aschendorff A, Laszig R, Beck R, Schild C, Kroeger S, Ihorst G, Wesarg T. Comparison of pseudobinaural hearing to real binaural hearing rehabilitation after cochlear implantation in patients with unilateral deafness and tinnitus. Otology & Neurotology 2010; 32:39-47

[21] Kleinjung T, Steffens T, Strutz J, Langguth B. Curing tinnitus with a cochlear implant in a patient with unilateral sudden deafness: a case report. Cases Journal 2009; 2:7462

[22] Van de Heyning P, Punte AK, De Bodt M, De Ridder D. Long-term tinnitus relief after cochlear implantation in single sided deafness. Presentation tinnitus conference 2011 Buffalo

[23] Van de Heyning P, Vermeire K, Diebl M, Nopp P, nderson I, De Ridder D. Incapacitating unilateral tinnitus in single-sided deafness treated by cochlear implantation. Annals of otology, rhinology & laryngology 2008;117(9):645-652

[24] Eggermont J.J, Roberts L.E. The neuroscience of tinnitus. Trends in Neuroscience 2004; Vol.27 No.11

[25] Rubinstein J, Tyler R.S, Johnson A, Brown C.J. Electrical suppression of tinnitus with high-rate pulse trains. Otology & Neurotology 2003;24:478-485

[26] Rothholtz VS, Tang Q, Wu EC, Fine EL, Djalilian H, Zeng F-G. Exploring the parametric space of tinnitus suppression in a patient with a cochlear implant. UCIrvine otolaryngology head & neck surgery; Poster presentation

[27] Dauman R, Tyler RS, Aran JM. Intracochlear electrical tinnitus reduction. Acta Otolaryngol (Stockh) 1993;113:291-295

[28] Van Wieringen A, Wouters J:LIST en LINT: sentences and numbers for quantifying speech understanding in severely impaired listeners for Flanders and The Netherlands. Int J Audiol 2008;47:348-355

[29] Andersson G. Tinnitus loudness matchings in relation to annoyance and grading of severety. Auris Nasus Larynx 2003 ;30 :129-133

[30] Quaranta N, Wagstaff S, Baguley DM. Tinnitus and cochlear implantation. International Journal of Audiology 2004 ;43 :245-251

[31] Figueiredo RR, De Azevedo AA, De Mello Oliveira P. Correlation analysis of the visual-analogue scale and the Tinnitus Handicap Inventory in tinnitus patients. Braz J Otorhinolaryngol 2009;75(1):76-9

[32] Meeus O, Blaivie C, Van de Heyning P. Validation of the Dutch and the French version of the Tinnitus Questionnaire. B-ENT 2007;3 Suppl 7:11-7

[33] Werkboek tinnitus. Stichting Nederlands Tinnitus Platform. ©NTP 2011; Versie 3.0 blz 41.

[34] Bouman TK, Luteijn F, Albersnagel FA, van der Ploeg FAE. Enige ervaringen met de Beck Depression Inventory. Gedrag – Tijdschrift voor psychologie. 1985;13:13-24

[35] Gatehouse S, Noble W. The speech, spatial and qualities of hearing scale (SSQ). International journal of audiology 2004;43:85-99

[36] http://www.nfu.nl/fileadmin/documents/Kwaliteitsborgingmensgebondenonderzoek.pdf
